# Dkk3 dependent transcriptional regulation controls age related skeletal muscle atrophy

**DOI:** 10.1038/s41467-018-04038-6

**Published:** 2018-05-01

**Authors:** Jie Yin, Lele Yang, Yangli Xie, Yan Liu, Sheng Li, Wenjun Yang, Bo Xu, Hongbin Ji, Lianghua Ding, Kun Wang, Gang Li, Lin Chen, Ping Hu

**Affiliations:** 1 State Key Laboratory of Cell Biology, CAS Center for Excellence in Molecular Cell Sciences, Shanghai Institute of Biochemistry and Cell Biology, Shanghai Institutes for Biological Sciences, Chinese Academy of Sciences, 320 Yueyang Road, 200031 Shanghai, China; 2 CAS Center for Excellence in Molecular Cell Science, Shanghai Institute of Biochemistry and Cell Biology, Chinese Academy of Sciences, University of Chinese Academy of Sciences, Shanghai, China; 3 Department of Rehabilitation Medicine, Center of Bone Metabolism and Repair, State Key Laboratory of Trauma, Burns and Combined Injury, Trauma Center, Research Institute of Surgery, Daping Hospital, The Third Military Medical University, 400042 Chongqing, China; 4 The 3rd Affiliated Hospital of Soochow University, Soochow University, 185 Jvqian Road, 21300 Changzhou, Jiangsu China; 5 Department of Neurology, East Hospital, Tongji University School of Medicine, 200120 Shanghai, China

## Abstract

Age-related muscle atrophy (sarcopenia) is the leading cause for disability in aged population, but the underlying molecular mechanisms are poorly understood. Here we identify a novel role for the secreted glycoprotein Dickkopf 3 (*Dkk3*) in sarcopenia. Forced expression of *Dkk3* in muscles in young mice leads to muscle atrophy. Conversely, reducing its expression in old muscles restores both muscle size and function. Dkk3 induces nuclear import of β-catenin and enhances its interaction with FoxO3, which in turn activates the transcription of E3 ubiquitin ligase *Fbxo32* and *Trim63*, driving muscle atrophy. These findings suggest that Dkk3 may be used as diagnostic marker and as therapeutic target for age-related muscle atrophy, and reveal a distinct transcriptional control of Fbxo32 and Trim63.

## Abstract (web summary)

Ageing is associated with muscle atrophy. Here, the authors show that the secreted glycoprotein Dickkopf 3 promotes muscle atrophy by inducing nuclear import of beta-catenin, its association with FoxO3 and the consequent activation of the atrophy-related genes *Atrogin1* and *MuRF1*.

## Introduction

Aging related decline of muscle mass and strength (sarcopenia) leads to limitation of mobility, metabolic changes, and mortality^1,2^. Although many molecules have been indicated to be associated with sarcopenia^1,2^, identification of factors present in circulation that correlate well with the markers of muscle atrophy is still challenging. Recently, it has been reported that reduction of circulating oxytocin is correlated with age. Oxytocin can facilitate myogenic progenitor cell proliferation to restore muscle regeneration in old mice, which in turn improves muscle functions^3^. Few circulating factors secreted by skeletal muscles and functioning directly on muscle mass maintenance have been identified to date.

The transcription activation of E3 ubiquitin ligase *Fbxo32* (*Atrogin1/MAFbx*) and *Trim63* (*MuRF1*) is a key regulatory step in diverse types of muscle atrophy^4,5^. Depletion of Trim63 helps muscle mass maintenance with age^6^. FoxO3, a member of Forkhead transcription family, is required for the transcription activation of both *Fbxo32* and *Trim63*^7,8^. FoxO3 has a central role in regulating muscle atrophy and it is sufficient to induce atrophy^9,10^. Many factors regulate skeletal muscle atrophy by modulating the activity of FoxO proteins^11^. IGF/PI3K/Akt signaling, IGF/mTOR signaling, and PGC-1α can all protect skeletal muscle from atrophy by inhibiting FoxO3 activity^8,12–16^. Wnt7a induces hypertrophy in both normal and DMD mice by direct activation of the Akt/mTOR pathway in an IGF receptor-independent manner^17,18^, suggesting that the non-canonical Wnt signaling pathway has important roles in muscle mass control.

Dkk3 is a secreted glycoprotein belonging to the Dickkopf (Dkk) family^19^. Dkk1, 2, and 4 can bind Wnt co-receptor Lrp5/6 and Kremen proteins to antagonize Wnt signaling^20–25^. Unlike other members in the Dkk family, Dkk3 is unable to bind Lrp and Kremen proteins^23,24,26^ and its receptor remains to be determined. The functions of Dkk3 in Wnt signaling pathway have not been fully understood. Dkk3 level is increased in circulating blood in aged population and its expression is upregulated during cellular senescence in prostate basal epithelial cells^27^, suggesting that Dkk3 may have a role in aging related disorders. Dkk3 has been suggested to function in myogenesis. In zebrafish, it increases the level of phosphorylated p38α and activates the transcription of *Myf5* through interaction with Integrin α6b^28,29^. The translation of Dkk3 is in turn inhibited by miR3906 that is encoded by the first intron of *Myf5*. This feedback loop regulates the embryonic myogenesis in zebrafish^30^. The functions of Dkk3 in muscle mass control remain to be elusive.

Whether sarcopenia is induced by molecules produced by muscle or from other organs is still under debating. Here we identified Dkk3 as the key secreted factor generated by muscles to induce sarcopenia and characterized the mechanism of Dkk3-dependent transcription activation of Fbxo32 and Trim63. These findings shed lights on understanding the causing of sarcopenia.

## Results

### *Dkk3* expression levels are increased in age-related muscle atrophy

**Fig. 1:**
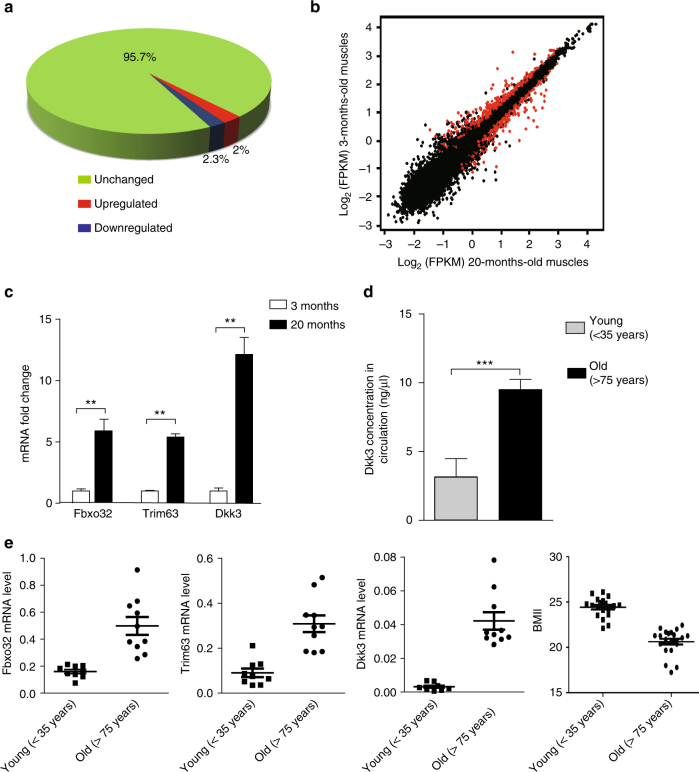
*Dkk3* is upregulated in muscles with age-related atrophy. **a** A pie diagram indicating the percentage of genes showing expression level changes in old versus young muscles. RNA was isolated from TA muscles showing atrophy and subjected for mRNA-sequencing analysis. The percentage of genes downregulated in TA muscles isolated from old mice compared to that in TA muscle isolated from young mice was indicated by blue. The percentage of genes upregulated in old TA muscles was indicated by red. The percentage of genes unchanged was indicated by green. **b** A scatter plot showing the comparison of transcriptome of old muscles showing atrophy versus young muscles. The red dots indicated the genes that were differentially expressed in old muscles versus young muscles. Statistic analysis was based on two independent mRNA-seq experiments for each sample. **c** Expression levels of *Fbxo32*, *Trim63*, and *Dkk3* in primary myofibers isolated from young or old mice, respectively. RT-qPCR was performed with RNA extracted from the primary myofibers. Error bars indicated standard deviation (s.d.) and were based on three independent experiments. ** indicated *p* < 0.01. **d** Dkk3 protein level was elevated in peripheral blood of sarcopenia patients. Dkk3 protein level was examined by ELISA in blood samples taken from sarcopenia patients and healthy young humans. Error bars indicated s.d. and were based on 20 samples. *** indicated *p* < 0.001. **e** *Dkk3* expression level was correlated with *Fbxo32* and *Trim63* expression level and BMI (body mass index) in aged human muscles. RT-qPCR assays were performed with muscle biopsies obtained from young or old patients and the results were normalized to GAPDH. The line indicated mean value. Error bars indicated s.d. and were based on 10 biological replicas. All *p-*values were based on two-tailed *t-*test

To identify the genes specifically upregulated or downregulated in aging related muscle atrophy (sarcopenia), tibialis anterior (TA) muscles from 3-month-old (Young) and 20-month-old (Old) mice were harvested. Mice at both ages (3-month-old and 20-month-old) were healthy. No tumor, obesity, injury, or other diseases were detected. Muscle fiber size was reduced in 20-month-old mice, suggesting the occurrence of muscle atrophy. RNA sequencing analysis was then performed with these muscle samples. Overall, 24,799 genes were detected. Among them, 95.7% maintained constant expression level in both young and old muscles; 2% (501) were significantly upregulated; 2.3% (584) were significantly downregulated (Fig. 1a, b) in old muscle. Spliceosome genes were enriched in the upregulated group and Parkinson’s disease-related genes were enriched in the downregulated group (Supplementary Fig. 1a). Consistent with previous reports, the expression level of muscle atrophy marker *Fbxo32* and *Trim63* were upregulated in old muscles for two- to threefolds (Supplementary Fig. 1b, c)^4,5^. We noticed that the expression level of *Dkk3* was also significantly increased in old muscles for about fourfolds (Supplementary Fig. 1b, c). Dkk3 is a secreted protein belonging to the Dickkopf family^19,31^, and is enriched in brain and muscle tissues (Supplementary Fig. 2a). As indicated by the RT-qPCR analysis, Dkk3 was the only Dkk family member enriched in muscle tissue (Supplementary Fig. 2b). It is the most divergent member of the Dickkopf family in both protein structure and functions^19^. It does not bind the common receptors shared by other Dkk family members and Wnt ligands^24,28,31^, and whether it regulates Wnt signaling pathway like the other members of Dkk family is controversial^24,25,28,32,33^. Its functions in muscle atrophy have not been characterized yet.

The upregulation of *Fbxo32*, *Trim23*, and *Dkk3* in old muscles was first confirmed by RT-qPCR with total RNA extracted from muscle tissues (Supplementary Fig. 3a). There were multiple lineages of cells in muscle tissue, such as blood cells, mesenchymal cells, and muscle cells. To further confirm the expression of *Fbxo32*, *Trim23*, and *Dkk3* in muscle cells, myofibers were isolated from either 3- or 20-month-old mouse muscles and RT-qPCR was performed. Expression levels of all three genes were markedly increased, suggesting the upregulation of these genes was enriched in muscle lineage cells (Fig. 1c; Supplementary Fig. 3b). The primary myofibers contain terminal differentiated muscle cells and a few satellite cells. We further compared the expression level of *Dkk3* in muscle satellite cells with that in myofibers. Dkk3 was barely detected in satellite cells, while its level was significantly higher in myofibers (Supplementary Fig. 3b), suggesting that terminal differentiated muscle cells were the major source of Dkk3.

Dkk3 is a secreted protein. We therefore investigated whether Dkk3 protein level in circulation was also upregulated in old mice by ELISA. The serum Dkk3 concentration was significantly higher in old mice (Supplementary Fig. 4), suggesting that Dkk3 could be a marker for sarcopenia diagnosis.

*Dkk3* is highly conserved across species (Supplementary Fig. 3c), we next examined the expression level of *Dkk3* in human muscle samples. Consistent with the observations in mice, *Fbxo32*, *Trim63*, and *Dkk3* were upregulated in muscles from aged patients with lower BMI (body mass index) as well (Fig. 1e). Furthermore, Dkk3 protein level in circulation was also elevated in the elders (Fig. 1d), that is consistent with previous reports^27^. These results revealed the correlated upregulation of Dkk3 with Fbxo32 and Trim63, suggesting high *Dkk3* expression level to be the potential marker for sarcopenia.

### Over-expression of *Dkk3* leads to muscle atrophy in young mice

**Fig. 2:**
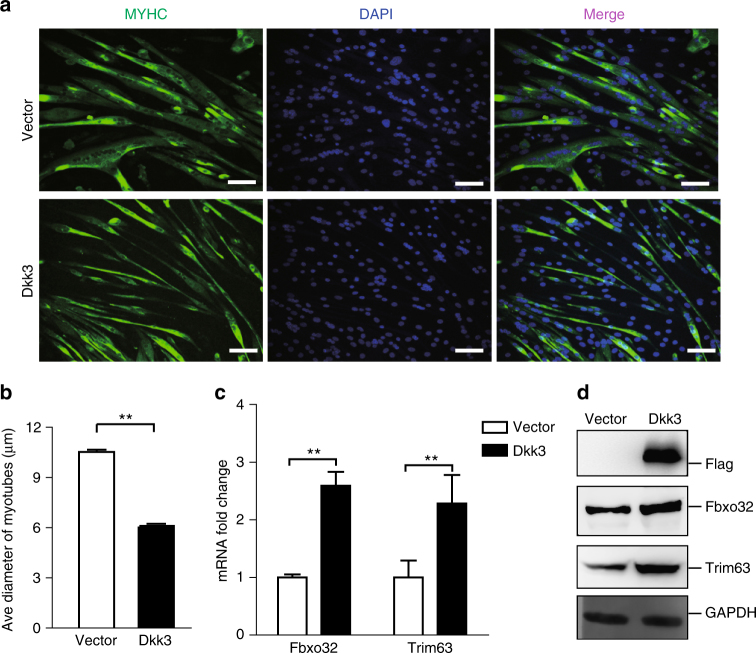
Over-expression of *Dkk3*-induced atrophy in cultured myotubes. **a** Representative images of myotubes over-expressing *Dkk3*. Primary myotubes were infected by adenovirus-encoding Dkk3 or control vector. Seventy two hours after infection, the myotubes over-expressing *Dkk3* were significantly thinner than those infected by control vector. Green indicated myosin heavy chain (MYHC) immunofluorescent staining. Blue indicated DAPI staining of nuclei; Merge indicated merged images of MYHC and DAPI. Scale bars, 50 μm. **b** Quantification of the average diameters of the myotubes over-expressing *Dkk3* or vector control. Error bars indicated s.d. and were based on three independent experiments. The diameters of 900 myotubes were measured in each experiment. ** indicated *p* < 0.01. **c** *Fbxo32* and *Trim63* expression levels increased after *Dkk3* over-expression. Vector indicated myotubes infected with adenovirus-encoding vector control. Dkk3 indicated myotubes infected with adenovirus-encoding Dkk3. Error bars indicated s.d. and were based on three independent experiments. ** indicated *p* < 0.01. **d** Over-expression of *Dkk3* increased the protein levels of Fbxo32 and Trim63. Immunoblotting assays were performed with whole-cell extracts from myotubes over-expressing *Dkk3* or vector control. Dkk3 was tagged with Flag tag. GAPDH served as internal control. All *p*-values were based on two-tailed *t-*test

To further investigate the functions of Dkk3 in muscle atrophy, we over-expressed *Dkk3* in myotubes. Myotubes differentiated from young primary myoblasts were infected by adenovirus-encoding *Dkk3* with Flag tag inserted after the signal peptide. Seventy two hours after infection, the myotubes were markedly thinner than those infected by vector virus (Fig. 2a, b). Consistent with the cell morphology changes, both the mRNA levels and the protein levels of *Fbxo32* and *Trim63* were significantly increased upon *Dkk3* over-expression (Fig. 2c, d). These results suggest that Dkk3 can induce atrophy in young myotubes.

As a secreted protein, Dkk3 should function both intracellularly and extracellularly. To investigate whether Dkk3 can function extracellularly, recombinant Dkk3 was purified (Supplementary Fig. 5a) and added to the medium to treat myotubes differentiated from primary myoblasts. An irrelevant secreted protein (Fab) was used as control. Forty-eight hours after Dkk3 treatment, the myotubes became significantly thinner than those in the control group (Supplementary Fig. 5b, c). The fusion index of myotubes remained unchanged, suggesting the thinner myotubes are not due to differentiation defects (Supplementary Fig. 5d). These results indicate the occurrence of myotube atrophy. Consistently, both the mRNA and protein levels of Fbxo32 and Trim63 were significantly upregulated upon extracellular Dkk3 treatment (Supplementary Fig. 5e, f). Together, these results suggest that Dkk3 works as an autocrine factor to induce muscle atrophy.

**Fig. 3:**
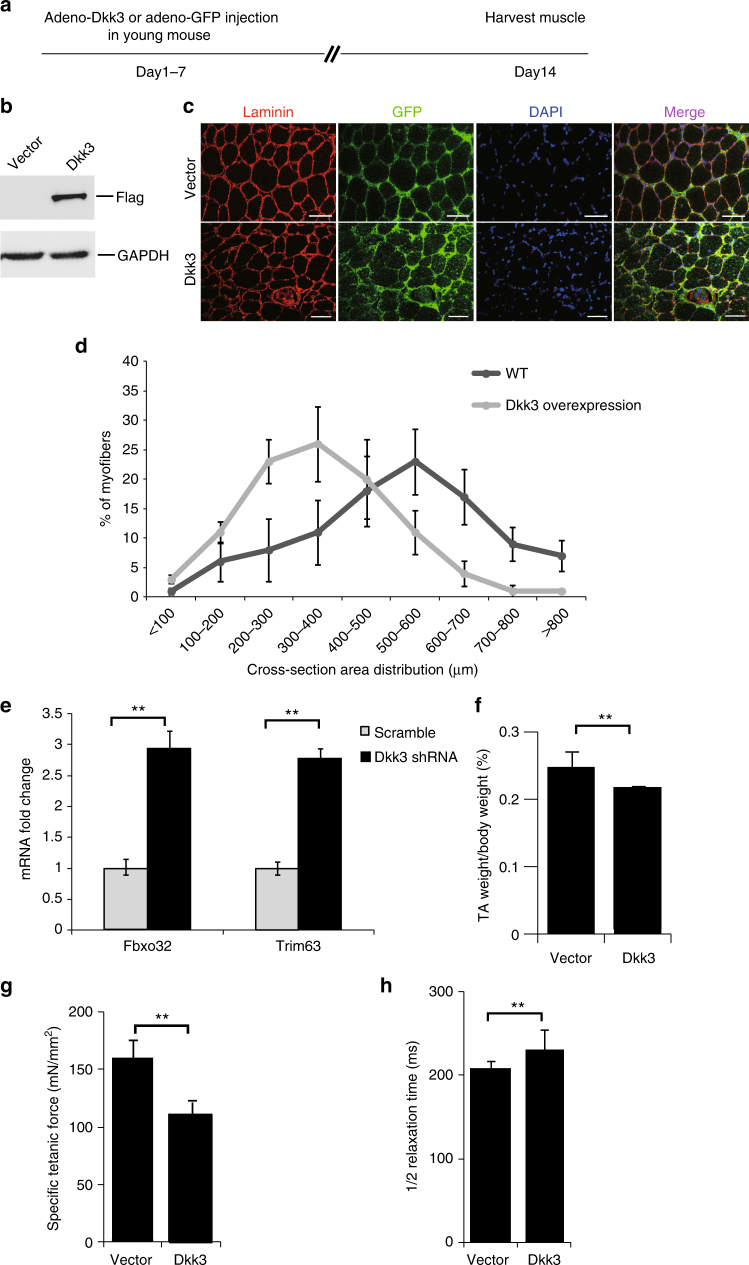
Over-expression of *Dkk3* in vivo induced muscle atrophy in young mice. **a** The schedule of virus injection. **b** Adenovirus-encoding Flag-Dkk3-IRES-GFP was injected to TA muscles in young mice (3 months) intramuscularly. Adenovirus-encoding GFP vector was injected to the TA muscle in mice with the same age and gender. Injections were performed once a day for 7 continuous days. TA muscles were harvested at day 14 for further analysis. A small portion of the TA muscles was used to make protein extracts. Anti-Flag immunoblotting was performed to detect the expression of the ectopic Flag-*Dkk3*. **c** Immunofluorescent staining images of muscle cross sections derived from TA muscles over-expressing *Dkk3* or vector control. Red indicated laminin staining; green indicated GFP; DAPI indicated nuclei; merge indicated merged images of laminin, GFP, and DAPI. Scale bars, 50 μm. **d** Percentage distribution of muscle fiber cross section area derived from muscles over-expressing *Dkk3* or GFP. The cross areas of all 1500 fibers in each TA muscle were checked. Black squares indicated muscle sections with ectopic *Dkk3* expression. Gray dots indicated muscle sections expressing vector. Error bars indicated s.d. and were based on five independent experiments. **e** *Fbxo32* and *Trim63* mRNA levels were measured by RT-qPCR in TA muscles with ectopic *Dkk3* or GFP expression. Error bars indicated s.d. and were based on five independent experiments. ** indicated *p* < 0.01. **f** Weight of TA muscles over-expressing *Dkk3* was compared to that expressing vector control. Error bars indicated s.d. and were based on five independent experiments. ** indicated *p* < 0.01. **fg** Specific titanic force of TA muscles over-expressing *Dkk3* or vector control in young mice (3 months). Error bars indicated s.d. and were based on 5 independent experiments. ** indicated p < 0.01. **h** 1/2 relaxation time of TA muscles over-expressing *Dkk3* or vector control in young mice (3 months). 1/2 relaxation time of TA muscles in old mice were compared to that of young mice (3 months). Error bars indicated s.d. and were based on five independent experiments. ** indicated *p* < 0.01. All *p*-values were based on two-tailed *t-*test

To further explore whether the elevated Dkk3 level could induce muscle atrophy in vivo, adenovirus-encoding *Flag*-tagged *Dkk3* and GFP was injected into the TA muscle of young mice (3-month-old) intramuscularly once a day for a week (Fig. 3a). Only adenovirus-expressing GFP was injected into the TA muscle of another young mouse with the same gender and age as the Dkk3-recipient mouse. Seven days after the final virus injection, TA muscles were harvested. The expression of *Flag-*tagged *Dkk3* was detected by immunoblotting (Fig. 3b). The muscle fiber size was also examined. The size of the muscle fibers with *Dkk3* over-expression was significantly smaller than that from muscle expressing GFP (Fig. 3c, d). Consistently, both the expression levels of *Fbxo32* and *Trim63* were significantly increased in TA muscles expressing ectopic Dkk3 as indicated by RT-qPCR and immunoblotting (Fig. 3e; Supplementary Fig. 6). The TA muscle weight was also decreased after *Dkk3* over-expression (Fig. 3f). The contraction abilities of TA muscle over-expressing *Dkk3* also decreased significantly (Fig. 3g), while the 1/2 relaxation time increased (Fig. 3h). These observations suggest that ectopic expression of *Dkk3* in young muscle is sufficient to induce muscle atrophy.

To rule out the variation among individuals, we also performed another set of experiments where virus encoding *Dkk3* was injected in TA muscle in one leg and virus encoding *GFP* was injected to TA muscle in the opposite leg. The virus was injected at the same frequency as described above in Fig. 3 (Supplementary Fig. 7a). Seven days after the final injection, TA muscles were harvested. Similar experiments were performed. The fiber size and TA muscle weight were all decreased (Supplementary Fig. 7b, c and e). The *Atrogin1* *Fbxo32* and *Trim63* mRNA levels were significantly upregulated (Supplementary Fig. 7d). The muscle contraction abilities were also impaired (Supplementary Fig. 7f, g).

### Reduction of Dkk3 level rescues aging related muscle atrophy

**Fig. 4:**
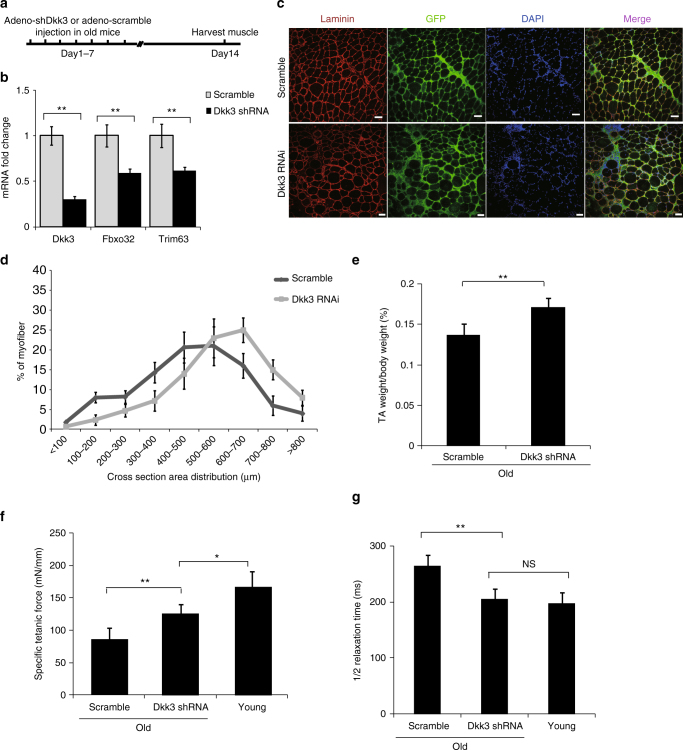
Reduction of Dkk3 level rescued age-related muscle atrophy. **a** Adenovirus-encoding shRNA against *Dkk3* was injected to TA muscles in old mice (20 months) intramuscularly. Adenovirus-encoding scramble shRNA was injected to the TA muscle in a mouse with the same age and gender as control. Both pieces of shRNA were co-expressed with GFP to label the infected muscle fibers in vivo. Injections were performed once a day for 7 continuous days. TA muscles were harvested at day 14 for further analysis. **b** Gene expression levels of *Dkk3*, *Fbxo32*, and *Trim63* in TA muscles injected with shRNA against *Dkk3* or scramble control as indicated by RT-qPCR. Error bars indicated s.d. and were based on five independent experiments. ** indicated *p* < 0.01. **c** Representative immunofluorescent staining images of muscle cross sections derived from TA muscles in old mice treated with shRNA against *Dkk3* or scramble control. The cross areas of all 1500 fibers in each TA muscle were checked. Red indicated laminin staining; green indicated GFP; DAPI indicated nuclei; merge indicated merged images of laminin, GFP, and DAPI staining. Scale bars, 50 μm. **d** Percentage distribution of muscle fiber cross section area derived from muscles treated with shRNA against *Dkk3* or scramble control. Black dots indicated muscle sections with scramble control treatment. Gray squares indicated muscle sections with *Dkk3* RNAi. **e** The percentage of TA muscle (by weight) in whole body muscles derived from mice treated with shRNA against *Dkk3* or scramble control. Error bars indicated s.d. and were based on five independent experiments. ** indicated *p* < 0.01. **f** Specific titanic force of TA muscles treated with shRNA against *Dkk3* or scramble control in old mice (20 months). The specific titanic force of TA muscles in old mice were compared to that of young mice (3 months). **g** 1/2 relaxation time of TA muscles treated with shRNA against *Dkk3* or scramble control in old mice (20 months). 1/2 relaxiation time of TA muscles in old mice were compared to that of young mice (3 months). All error bars indicated s.d. based on 5 independent experiments. ** indicated *p* < 0.01. * indicated *p* < 0.1. NS indicated no significant changes. All *p*-values were based on two-tailed *t*-test

We next investigated whether reducing *Dkk3* expression level could rescue muscle functions in old muscles. Adenovirus-encoding shRNA against *Dkk3* was injected intramuscularly to TA muscle in 20-month-old mice (Fig. 4a). Adenovirus-encoding scramble shRNA was injected to the TA muscle of a mouse with the same age and gender. The shRNA reduced Dkk3 expression level efficiently (Fig. 4b). The expression levels of *Fbxo32* and *Trim63* were downregulated significantly upon Dkk3 RNAi (Fig. 4b). The fiber size of TA muscle was increased markedly after Dkk3 RNAi compared to that of TA muscle with scramble shRNA injection (Fig. 4c, d).

We further investigated whether the contraction abilities of the old muscles subjected to Dkk3 RNAi were also improved. The weight of TA muscles increased after Dkk3 RNAi (Fig. 4e). The specific titanic force was also increased after Dkk3 RNAi in old mice, close to that of young mice (Fig. 4f). The 1/2 relaxation time of the old TA muscles treated with shRNA against Dkk3 was also improved to the level comparable to that of young TA muscles (Fig. 4g). Together, these results reveal that reduction of Dkk3 level in old mice can improve both muscle fiber size and functions, suggesting Dkk3 to be a potential target to treat sarcopenia.

To avoid the variation among individuals, we also performed temporary RNAi knockdown experiments with shRNA and scramble control virus injected to TA muscles at the opposite side of the same mouse (Supplementary Fig. 8a). Similar to the above set of experiment, the expression levels of Fbxo32 and Trim63 were downregulated significantly when *Dkk3* expression was reduced by RNAi (Supplementary Fig. 8b). Consistent with the above results, both the fiber size and muscle functions were improved after Dkk3 RNAi (Supplementary Fig. 8c–g). Together, these results suggest that reduction of Dkk3 level in old muscle can improve muscle functions.

### Muscle atrophy induced by starvation and cachexia is Dkk3 independent

**Fig. 5:**
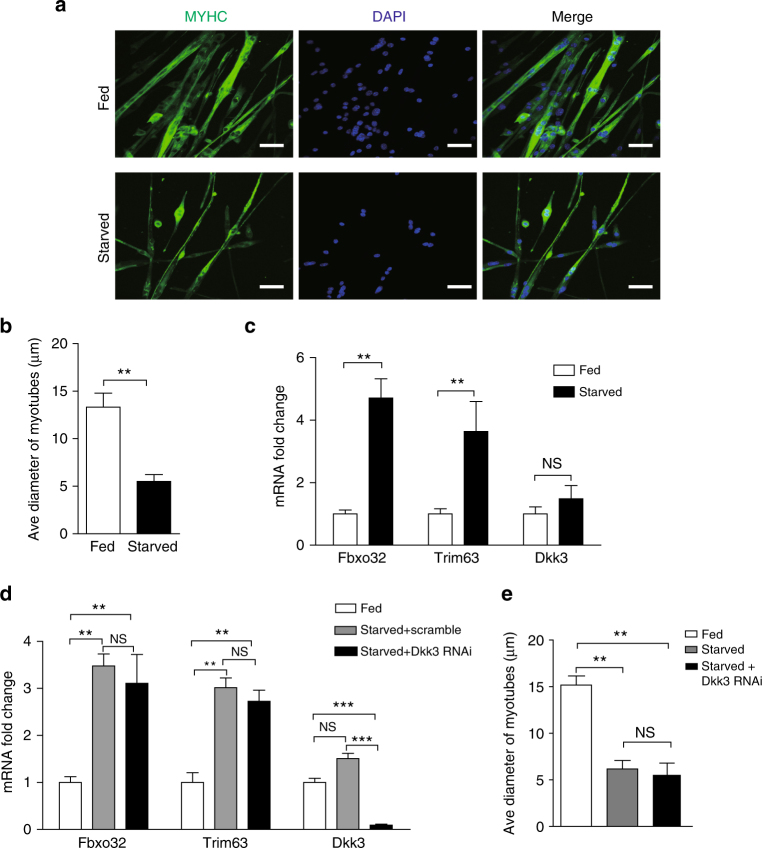
Starvation-induced muscle atrophy did not depend on Dkk3. **a** Representative images of atrophy induced by starvation in myotubes. Green indicated MYHC staining; blue indicated DAPI staining of nuclei; Merge indicated merged images of MYHC and DAPI. Scale bars, 50 μm. **b** Average diameters of myotubes with or without serum starvation. Error bars indicated s.d. and were based on three independent experiments. ** indicated *p* < 0.01. **c** Expression levels of *Fbxo32*, *Trim63*, and *Dkk3* in fed or starved myotubes. Error bars indicated s.d. and were based on three independent experiments. ** indicated *p* < 0.01. NS indicated no significant changes. **d** *Dkk3* RNAi showed no effects on *Fbxo32* and *Trim63* expression level in starved myotubes. Primary myotubes were first serum starved in Hank’s buffer. Adenovirus-encoding shRNA against *Dkk3* or scramble control was next applied to the starved myotubes. Forty-eight hours after virus infection, the myotubes were harvested and subjected for RT-qPCR analysis of the mRNA levels of *Fbxo32, Trim63,*and *Dkk3*. Error bars indicated s.d. and were based on three independent experiments. ** indicated *p* < 0.01. NS indicated no significant changes. **e** Average diameters of myotubes with or without serum starvation, and *Dkk3* RNAi after serum starvation. Error bars indicated by s.d. and were based on three independent experiments. ** indicated *p* < 0.01. NS indicated no significant changes. All *p*-values were based on two-tailed *t*-test

We next investigated whether Dkk3 also functions in other types of muscle atrophy. Starvation-induced muscle atrophy is a widely used atrophy model^7,10,34^. Primary myotubes were serum starved in Hank’s buffered salt solution for 24 h to induce atrophy. The myotubes were significantly thinner after serum starvation (Fig. 5a, b), suggesting the occurrence of atrophy. Consistent with previous reports^7,10,34^, Fbxo32 and Trim63 level increased markedly (Fig. 5c). In sharp contrast to the scenario in aging related muscle atrophy, *Dkk3* expression level remained unchanged after serum starvation (Fig. 5c). We further performed *Dkk3* RNAi in myotubes with starvation-induced atrophy. *Dkk3* expression level was reduced efficiently by the shRNA treatment. In contrast to the observations in aging related muscle atrophy, reduction of *Dkk3* expression level in starved myotubes had no effect on *Fbxo32* and *Trim63* expression levels (Fig. 5d), and failed to rescue the fiber size reduction after starvation (Fig. 5e). These observations suggest that not all types of muscle atrophy are Dkk3 dependent.

We also examined the expression level of *Dkk3* in cachetic muscle induced by lung cancer^35^. The average weight of gastrocnemius was reduced by about 20%, suggesting the occurrence of cachexia. Though the expression levels of *Fbxo32* and *Trim63* increased significantly, *Dkk3* expression remained unchanged (Supplementary Fig. 9). Together, these results suggest that upregulation of Dkk3 is not a universal feature for all types of muscle atrophy.

### Dkk3 induces recruitment of β-catenin at FoxO3 binding sites on Fbxo32 and Trim63 promoters

**Fig. 6:**
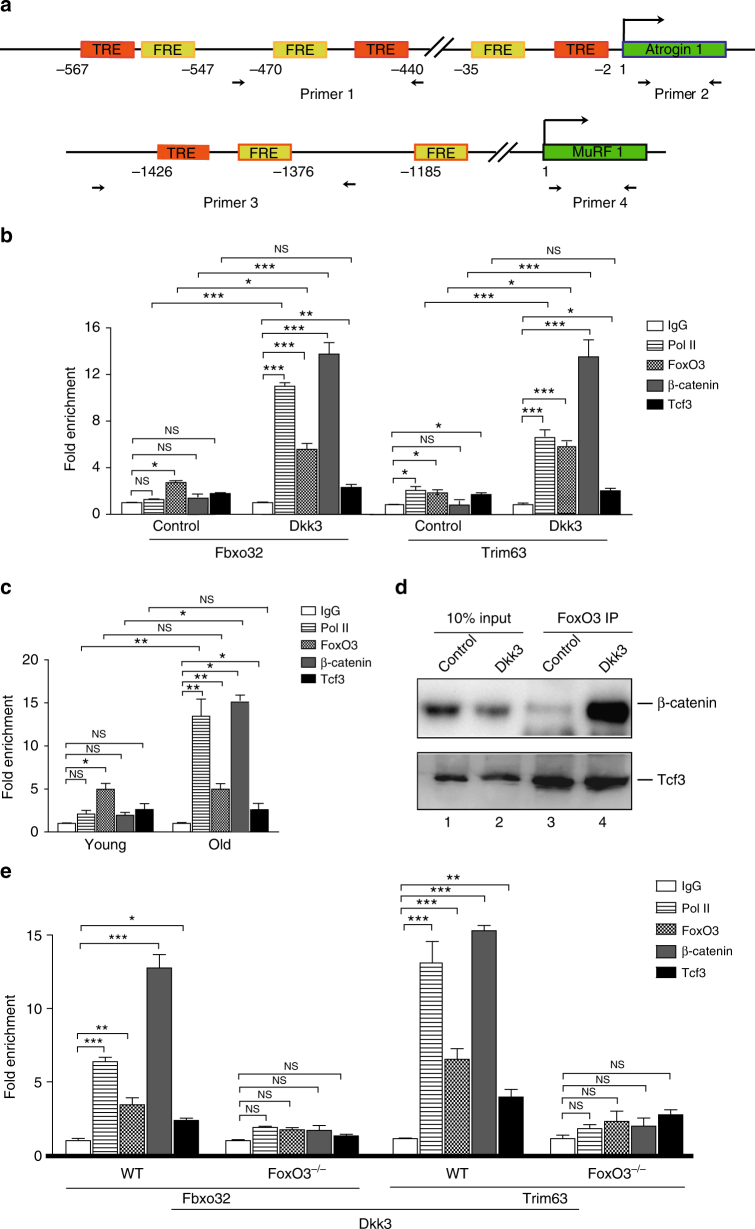
Dkk3-induced FoxO3-dependent recruitment of β-catenin on the core promoters of *Fbxo32* and *Trim63*. **a** Schematic illustration of the core promoter structures of *Fbxo32* and *Trim63*. **b** ChIP analysis of RNA pol II, FoxO3, β-catenin, and Tcf3 on *Fbxo32* and *Trim63* promoters in primary myotubes treated with Dkk3 or control protein. Error bars indicated s.d. and were based on four independent experiments. * indicated *p* < 0.1. ** indicated *p* < 0.01. *** indicated *p* < 0.001. NS indicated no significant changes. **c** ChIP analysis of RNA pol II, FoxO3, β-catenin, and Tcf3 on core promoters of *Fbxo32* and *Trim63* in muscle tissues isolated from young adult (3 months) or aged (20 months) mice. Error bars indicated s.d. and were based on five independent experiments. * indicated *p* < 0.1. ** indicated *p* < 0.01. *** indicated *p* < 0.001. NS indicated no significant changes. **d** Nuclear extracts prepared from myotubes treated with Dkk3 or control protein were subjected to immunoprecipitation with FoxO3 antibody. The immunoprecipitated proteins were subjected to immunoblotting with β-catenin and Tcf3. Lane 1 and 2 were 10% of input nuclear extracts. Lane 3 and 4 represented immunoprecipitation with FoxO3 antibody. **e** ChIP analysis of RNA pol II, FoxO3, β-catenin, and Tcf3 on the core promoters of *Fbxo32* and *Trim63* in *FoxO3* null TA muscles with or without Dkk3 over-expression. Error bars indicated s.d. and were based on five independent experiments. * indicated *p* < 0.1. ** indicated *p* < 0.01. *** indicated *p* < 0.001. NS indicated no significant changes. All *p*-values were based on two-tailed *t*-test

To further investigate the mechanism of *Fbxo32* and *Trim63* activation induced by Dkk3, we analyzed the structure of the core promoter of *Fbxo32* and *Trim63*. As reported previously, there were multiple FoxO3 recognition elements (FREs) in the core promoters of both *Fbxo32* and *Trim63*^7,8,12^. Further sequence analysis revealed that several FRE sites were paired with the adjacent Tcf recognition elements (TRE) within the core promoter regions of both genes (Fig. 6a). The promoter structures imply that Tcf transcription factors may work together with FoxO3 to activate transcription of *Fbxo32* and *Trim63*. We therefore performed chromatin immunoprecipitation (ChIP) assays to investigate the promoter occupation of FoxO3, Tcf3, β-catenin (Tcf3 co-activator), and RNA polymerase II (Pol II) in primary myotubes treated with Dkk3. Consistent with the upregulation of *Fbxo32* and *Trim63* expression observed above, Dkk3 treatment led to significant increase of RNA Pol II recruitment on both *Fbxo32* and *Trim63* genes (Fig. 6b), confirming the active transcription of these genes. As reported previously^7,10,12,36^, FoxO3 was detected on both *Fbxo32* and *Trim63* promoters. However, the FoxO3 occupation on *Fbxo32* and *Trim63* promoters showed only marginal increase upon Dkk3 treatment (Fig. 6b). In sharp contrast, markedly higher amount of FoxO3 was recruited to *Fbxo32* and *Trim63* promoters in starvation-induced atrophy (Fig. 6c). Low amount of Tcf3 was also detected on both *Fbxo32* and *MuRF 1* promoters and the amount of this protein bound on both promoters did not change after Dkk3 treatment (Fig. 6b). β-catenin could be barely detected on *Fbxo32* and *Trim63* promoters in normal primary myotubes. Surprisingly, the amount of β-catenin bound on *Fbxo32* and *Trim63* promoters markedly elevated in primary myotubes treated with Dkk3 (Fig. 6b). In sharp contrast, no β-catenin occupation on *Fbxo32* and *Trim63* promoters was detected in starvation-induced muscle atrophy (Supplementary Fig. 10). These results suggest the co-occupation of FoxO3, Tcf3, and β-catenin on *Fbxo32* and *Trim63* promoters in a Dkk3-dependent manner.

To further confirm the above observations in vivo, ChIP assays were performed in muscle tissues harvested from old (20-months-old) mice showing muscle atrophy. Consistently, significantly higher amount of β-catenin was recruited to *Fbxo32* and *Trim63* promoters in old muscles; while the amount of FoxO3 bound on *Fbxo32* and *Trim63* promoters only increased slightly (Fig. 6c); while fairly low amount of Tcf3 was recruited to the promoters of *Fbxo32* and *Trim63* (Fig. 6c). These results suggest that the recruitment of FoxO3 and β-catenin is the key to activate *Fbxo32* and *Trim63* transcription in sarcopenia. In sharp contrast, β-catenin was not required for *Fbxo32* and *Trim63* transcription activation in starvation-induced muscle atrophy (Supplementary Fig. 10). These results combined suggest that *Fbxo32* and *Trim63* transcription can be activated by distinct combination of activators and co-activators in response to different environmental cues.

It has been reported that FoxO proteins can compete with Tcf proteins to win β-catenin binding under oxidative stress and antagonize Wnt signaling pathway^37–39^. We then set out to examine whether the enhanced recruitment of β-catenin on *Fbxo32* and *Trim63* promoters antagonizes the transcription of canonical Wnt target genes. The mRNA level of the canonical Wnt targets *axin2* and *Ccnd1* remained the same in old muscles (Supplementary Fig. 11a). Consistently, the amount of promoter bound β-catenin on *Axin 2* and *Ccnd1* remained unchanged as well in muscles from young or old mice (Supplementary Fig. 11b). These results indicate that the increased β-catenin binding on the *Fbxo32* and *Trim63* promoters does not antagonize canonical Wnt signaling and there is no apparent competition of β-catenin between the promoters of atrophy-related genes and the canonical Wnt targets, suggesting that Dkk3-induced muscle atrophy is not dependent on canonical Wnt signaling. It is consistent with the previous reports that Dkk3 did not share common receptor with other Dkk family members and was not directly involved in Wnt signaling^23,24,26^. These results also hint an increase of the total amount of nuclei localized β-catenin by Dkk3 treatment. Indeed, the amount of nuclear β-catenin was higher in primary myofibers treated by Dkk3; while the amount of nuclei localized FoxO3 remained constant (Supplementary Fig. 12a, b).

We next investigated whether the Dkk3-induced co-occupation on *Fbxo32* and *Trim63* promoters by FoxO3, Tcf3, and β-catenin is mediated by protein–protein interactions. Nuclear extracts of primary myotubes treated by Dkk3 or control protein were subjected to immunoprecipitation with FoxO3 antibody. Significantly higher amount of β-catenin was detected to interact with FoxO3 after Dkk3 treatment (Fig. 6d). These results suggest that Dkk3 induces more β-catenin to be imported to nuclei to enhance β-catenin–FoxO3 interaction. FoxO3 further recruits β-catenin to *Fbxo32* and *Trim63* promoters through protein–protein interaction to activate *Fbxo32* and *Trim63* transcription in the presence of high dosage of Dkk3.

We then examined whether Dkk3 can affect other signaling pathways through β-catenin. Akt/mTOR and PCP pathways have been suggested to be regulated by non-canonical Wnt signaling in muscle cells^17,18,40^. We therefore examined whether Dkk3 affects Akt/mTOR and PCP signaling. In old muscles, Akt/mTOR was repressed (Supplementary Fig. 13a). When Dkk3 was over-expressed in myotubes either in vitro or in vivo, Akt/mTOR was also repressed, mimicking the phenomenon in old muscles (Supplementary Fig. 13b, c). These results are consistent with previous reports that activation of Akt and mTOR signaling can protect skeletal muscles from atrophy by inhibiting FoxO3^8,15,16,41^. Furthermore, the expression level of Akt was slightly downregulated by Dkk3 treatment. This observation indicates that Dkk3 related signaling may directly alter the expression level of Akt in addition to regulation of its phosphorylation level. RhoA was downregulated by Dkk3 over-expression and, to a less extent, in aged muscles (Supplementary Fig. 13). The Rac1 expression seems to be regulated by more complicated mechanism and cannot be repressed by Dkk3 (Supplementary Fig. 13). Together, these results suggest that Dkk3 can induce the inhibition of Akt/mTOR signaling though the canonical Wnt signaling is not affected.

We next examined whether β-catenin is required for Dkk3-dependent muscle atrophy. We infected myotubes differentiated from primary myoblasts with adenovirus-encoding shRNA against β-catenin and checked myotube diameters and expression levels of *Fbxo32* and *Trim63*. When β-catenin was depleted, Dkk3 was unable to induce the activation of *Fbxo32* and *Trim63* transcription (Supplementary Fig. 14c). Dkk3 was unable to induce muscle atrophy when β-catenin was knocked down (Supplementary Fig. 14a, b), suggesting that β-catenin is required for Dkk3-induced muscle atrophy.

We further investigated whether FoxO3 is essential for *Fbxo32* and *Trim63* transcription activation in Dkk3-induced muscle atrophy. Adenovirus-encoding Dkk3 was injected muscularly to the TA muscle of 3-month-old FoxO3^−^^/−^ mice. Adenovirus-encoding vector was injected to the TA muscle at the opposite side as control. In sharp contrast to Dkk3 over-expression in wild-type young mice (Fig. 3a, b), no fiber size change was observed after Dkk3 over-expression (Supplementary Fig. 12b, c). Furthermore, primary myotubes were isolated from wild-type or FoxO3 null mice and subjected for Dkk3 treatment. In sharp contrast to the observations in wild-type myotubes, Dkk3 lost the ability to induce the recruitment of β-catenin to *Fbxo32* and *Trim63* promoters when FoxO3 was depleted (Fig. 6e), suggesting that FoxO3 is required for the recruitment of β-catenin to the promoters of *Fbxo32* and *Trim63*.

All together, these results suggest that *Fbxo32* and *Trim63* transcription is activated by the combination of FoxO3 and β-catenin in a Dkk3-dependent manner specifically in aging related muscle atrophy.

## Discussion

In summary, we showed that Dkk3 level in circulation could indicate the occurrence of sarcopenia. Knocking down *Dkk3* in sarcopenia muscles could rescue not only the muscle fiber size but also the muscle contraction abilities. These results suggest that Dkk3 is a potential target to diagnose and treat sarcopenia.

The molecular mechanism of sarcopenia has not been fully understood and is under continuous active exploration. The activation of *Fbxo32* and *Trim63* transcription is the common key regulatory step shared by various types of muscle atrophy^4,5,42^. FoxO3 has been shown to be required for the transcription activation of *Fbxo32* and *Trim63*^7,8,12,43^. However, the mechanism of FoxO3-dependent *Fbxo32* and *Trim63* activation remains to be fully understood. Our results suggest that the activation of the *Fbxo32* and *Trim63* transcription can be achieved by different combinations of activators and co-activators in response to distinct atrophy stimuli. Although FoxO3 serves as the common key activator in *Fbxo32* and *Trim63* transcription activation, it takes different paths to accomplish the goal in response to distinct environmental cues. For example, in response to starvation, transcription activation of *Fbxo32* and *Trim63* is achieved by recruiting increasing amount of FoxO3 to the promoters of these genes; while in response to aging, FoxO3 activates *Fbxo32* and *Trim63* transcription by recruiting co-activator β-catenin. Furthermore, the choice of FoxO3-β-catenin combination is dependent on high dosage of Dkk3. Whether FoxO3 can work with other activator partner/co-activator to switch on *Fbxo32* and *Trim63* transcription and what signals FoxO3 to choose different partners to achieve the same goal are interesting questions for further exploration. Answering these questions will not only deepen our understanding on the mechanism of muscle atrophy triggered by different environmental cues, but also shed lights on the more general transcription regulation mechanism governing cell responses to distinct environmental cues.

Previous reports have shown that FoxO3 can compete with Tcf proteins to win β-catenin binding in response to oxidative stress^37–39^. Our findings suggest that FoxO3 and β-catenin can interact in the context of aging related muscle atrophy, but the interaction does not antagonize canonical Wnt signaling pathway. Even though Dkk1 is a well known Wnt signaling antagonist^19,20,31^, the role of Dkk3 in Wnt signaling is still under debate and poorly understood. Unlike other Dkk family members, Dkk3 can bind neither Wnt receptor Lrp6 nor the co-receptor Kremen proteins^23,24,26^. Dkk3 is unable to regulate Wnt signaling pathway under many circumstances^23,24,32,44^. However, it has also been reported to inhibit Wnt signaling in some cell types^45,46^. Our results suggest that Dkk3 has the ability to promote nuclear localization of β-catenin and its interaction to FoxO3, indicating that Dkk3 could regulate Wnt signaling in a FoxO3-dependent aspect. FoxO3 could prevent the nuclear localized β-catenin from being recruited to canonical Wnt target genes. Even though in the context of sarcopenia, FoxO3 only recruits the newly imported β-catenin in nuclei and does not affect β-catenin binding on canonical targets, high dosage of FoxO3 may further compete β-catenin away from canonical Wnt targets and inhibit Wnt signaling. This mechanism suggests that the context-dependent functions of Dkk3 in Wnt signaling regulation may be determined by FoxO3.

In the context of sarcopenia, Dkk3 treatment does not affect canonical Wnt signaling. In sharp contrast, Akt/mTOR and PCP signaling, which have been suggested to be regulated by non-canonical Wnt signaling in muscle cells^17,18,40^, were inhibited. These results are consistent with previous reports that activation of Akt signaling pathway by non-canonical Wnt leads to muscle hypertrophy. Together, these results reveal the critical role of non-canoical Wnt in muscle mass maintenance. Recent reports have suggested that non-canonical Wnt signaling is also an important regulator for muscle regeneration^47–49^. It will be interesting to explore the role of Dkk3 in muscle regeneration.

Dkk3 has also been reported to inhibit TGFβ signaling in both *Xenopus* and several mammalian cell types such as prostate epithelial cells, cervical cancers, and cartilage cells^50,51^. β-catenin has been implicated to be involved in Dkk3 mediated TGFβ repression^52^. How β-catenin bridges Dkk3-induced inhibition of non-canonical Wnt and TGFβ signaling is an interesting question for further exploration.

Elevated Dkk3 level has been indicated in many diseases. For example, Dkk3 was secreted in the urine of mice in stress induced tubular atrophy and fibrosis in chronic kidney diseases. Reducing Dkk3 protein level by mutation or antibody blockage can alleviate renal fibrosis and improve kidney functions^53^. Muscle is another type of tissue where high dosage of Dkk3 leads to degeneration of the functions. The identification of Dkk3 as the key regulator in aging related muscle atrophy has important therapeutic implications. Dkk3 could serve as a diagnosis marker and therapeutic target for sarcopenia.

## Methods

### Human samples

Muscle biopsies from 20 young adults (<35 years) and 20 geriatric (>70 years) humans were obtained from vastus lateralis under local anesthesia. Fat, nerve, and other non-muscle tissues were trimmed off. Circulating blood samples were taken from 20 young adults (<35 years) and 20 geriatric (>70 years) humans. All subjects and samples were collected according to protocols approved by the institutional review board and ethics committee of Daping Hospital, Third Military Medical University and the methods were carried out in accordance with the approved guidelines. Informed consent was obtained from all subjects. This project received Ethics Approval from the Medical Ethics Committee of Daping Hospital, Third Militry medical University.

### Statistics

We used the following formula described previously^54^ to determine the human sample size:

1 $$n = 1 + 2C\left( {\frac{s}{d}} \right)^2,$$

*n* indicated sample number; *s* indicated s.d.; *d* indicated difference between the two groups being compared. *C* was a constant. With a power of 90% and significance level of 95%, *C* = 10.51. We set to be able to detect all differences ≥60% of the s.d., therefore *s*/*d* = 0.6, *n* = 8.56. We determined to take the sample size of 10 for each group based on the above calculation.

All *p-*values were calculated based on two-tailed *t*-test.

### mRNA-seq and analysis

TA muscle was isolated from C57BL/6 young mice (3 months) or old mice (20 months). RNA was extracted by TRIZOL (Sigma) and processed for mRNA-seq by TruSeq Stranded mRNA sample prep kit (Illumina) according to the manufacturer’s instructions. The sequencing data were collected by HiSeq 3000 (Illumina). After obtaining the raw RNA-seq reads, we first trimmed the sequencing adaptors as well as low quality base pairs, then removed the PCR duplicated reads using in-house programs. The preprocessed reads were then aligned to the mouse reference genome (NCBI 37/UCSC mm10) using Tophat2 (version 2.0.10) guided by the RefSeq genes and allowing a maximum of two mismatches. Transcriptome profiling was thereafter performed using Cufflinks (version 2.1.1) against RefSeq genes for each sample. Differentially expressed genes were detected using Cuffdiff (version 2.1.1) against RefSeq genes.

Gene ontology (GO) Biological Process (BP) together with KEGG pathway databases were used for identification of functional enrichment among gene groups.

### Gene expression analysis

Total RNA was isolated using RNeasy kits (Qiagen). Overall, 1 μg of total RNA from each sample was reverse transcribed to cDNA using MuLV transcriptase (NEB) and oligo dT primer according to the manufacturer’s instruction. Briefly, RNA was first denatured at 85 °C for 3 min. MuLV reverse transcriptase was then added and incubated at 42 °C for 1 h. Quantitative PCR (qPCR) reactions were performed in triplicates using SYBR Green PCR master mix (DBI) in BioRad thermocycler system (BioRad) and analyzed by iQ5 optical system software (BioRad). The primers for RT-qPCR are listed below:

Mouse *Dkk3*-F: 5′-TGAGGCAGTGGCTACACAAG-3′,

Mouse *Dkk3*-R: 5′-GCTGGTATGGGGTTGAGAGA-3′,

Mouse *Fbxo32*-F:5′-AGAGAGGCAGATTCGCAAGCGT-3′,

Mouse *Fbxo32*-R: 5′-TGCAAAGCTGCAGGGTGACCC-3′,

Mouse *GAPDH*-F: 5′-ACCCAGAAGACTGTGGATGG-3′,

Mouse *GAPDH*-R: 5′-ACACATTGGGGGTAGGAACA-3′,

Human *DKK3*-F: 5′-TTCATCCAGCAGTGTTGCTC-3′,

Human *DKK3*-R: 5′-GGTGTGGGGTAGTGGAGAGA-3′,

Human *FBXO32*-F: 5′-GGCTGCTGTGGAAGAAACTC-3′,

Human *FBXO32*-R: 5′-TGTGACAGTGTTTGCAGAGC-3′,

Mouse *Trim63*-F: 5′-ACCTGCTGGTGGAAAACATC-3′,

Mouse *Trim63*-R: 5′-CTTCGTGTTCCTTGCACATC-3′,

Human TRIM63-F: 5′-GACATTGGCAATGACGCTCA-3′

Human TRIM63-R: 5′-TCACATCTAAATGATCTTCTTAA-3′

Human *GAPDH*-F: 5′-GAGTCAACGGATTTGGTCGT-3′

Human *GAPDH*-R: 5′-TTGATTTTGGAGGGATCTCG-3′

### Primary myofiber isolation and culture

The protocol to isolate primary myofibers was kindly provided by Dr. Rudnicki (University of Ottawa) and as described previously^49,55^. Briefly, EDL muscles were isolated form tendon to tendon. The isolated EDL was digested in DMEM medium (Invitrogen) contain 0.2% collagenase D (Roche) for 90 min and myofibers were disassociated. The digested muscle tissues were triturate three times with a wide bore 5 ml pipet. Let the tube sit at room temperature for 5 min to allow fibers to settle at the bottom of the tube. Use 10 ml or 5 ml pipet to remove the supernatant. Wash the triturated fibers with pre-warmed DMEM medium twice. Pick out single fibers with wide bore yellow tips and transfer to tubes containing Trizol lysis buffer. About 200 myofibers were collected for each RT-qPCR. The isolated young myofibers were cultured under the condition described previously^56,57^. Briefly, myofibers were cultured in DMEM medium containing 20% FBS (HyClone), 1% chicken embryo extracts (Accurate Chemicals) in dishes coated with serum.

### Cell culture and differentiation

Primary myoblasts were isolated as previously described^58^. The primary myoblasts were cultured in collagen-coated dishes in F10 basal medium (Invitrogen) containing 10% FBS and 2.5 ng/ml FGF (Invitrogen). The cells were then differentiated in differentiation medium (DMEM medium (invitrogen) containing 2% horse serum (HyClone)) for 2 days. C2C12 cells (ATCC) were cultured in DMEM medium (Invitrogen) containing 10% FBS (HyClone), and differentiated in DMEM medium (Invitrogen) containing 2% horse serum (HyClone) for 3 days. The differentiated myotubes were further isolated by pre-attaching to plates for three times. Horse serum was removed from the differentiation medium when serum starvation was performed.

### Animals

C57BL/6 mice (Charles River) >20 months were utilized as the aged mouse model. C57BL/6 mice (Charles River) 3 months were utilized as the young mouse model. Sex of the mice was randomly selected. Animal care and use were in accordance with the guidelines of the Shanghai Institute of Biochemistry and Cell Biology, Chinese Academy of Sciences. Permission to perform animal experiments was granted by the animal ethical committee of Shanghai Institute of Biochemistry and Cell Biology, Chinese Academy of Sciences. Mice within each sample group were selected randomly. In each group, five independent mice were used, and data from all animal samples were collected and subjected for statistic analysis.

The lung cancer cachexia model was obtained from Dr. Hongbin Ji in Shanghai Institute of Biochemistry and Cell Biology, Chinese Academy of Sciences. The generation of the lung cancer model has been described previously^35,59^. Briefly, lung tumor was induced in 8 weeks old KrasG12D Trp53lox/lox mice by nasal inhalation of adenovirus carrying Cre (5 × 106 p.f.u.). The tumor was given 8 weeks to progress. Compared to the control mice without tumor, the body weight of mice carrying lung cancer decreased 8.9%. Gastrocnemius muscles were harvested for immunoblotting assays.

### Muscle virus injection

Adenovirus-encoding *Dkk3* or *GFP* was injected into the TA muscles of 3-month-old C57BL/6 (Jackson Laboratory) mice. 50 µl of adenovirus-encoding Dkk3 (1.3 × 107 unit/µl) or GFP (1.3 × 107 unit/µl) was injected into TA muscle once a day for 7 continuous days. TA muscles were harvested 14 days after the last injection. Overall, 50 µl of adenovirus-encoding shRNA against Dkk3 (1.3 × 10^7^ unit/µl) or scramble RNA (1.3 × 10^7^ unit/µl) was injected into TA muscles of 20-month-old C57BL/6 mice once a day for 7 continuous days. TA muscles were harvested 14 days after the last injection. For each type of virus, 5 mice were injected.

### In vivo muscle force analysis

In vivo TA muscle force analysis was performed with 1300 A 3-in-1 whole animal system (Aurora Scientific) as previously described^60–62^. Mice were anesthetized by 3-bromo-2-fluoro-phenyl methanol (31.2 g/kg body weight) injection and kept warm by heat lamp. The hind limb was shaved and immobilized by fixing the leg in a frame without disturbing the blood flow. Tie around the patella ligament of the distal TA muscle using a bread silk suture. Make an incision to expose the sciatic nerve and tie another knot around the proximal end of the sciatic nerve. Attach the distal TA tendon suture loop to the lever arm hook of the instrument and measure the contractile force. During the process, TA muscle was constantly superfused with pre-warmed 37 °C Ringer’s buffer (118 mM NaCl, 4.75 mM KCl, 1.18 mM KH_2_PO_4_, 1.18 mM MgSO_4_, 2.5 mM CaCl_2_, 25 mM NaHCO_3_, and 11 mM glucose). The results were analyzed by DMA software (Aurora scientific). For each treatment, five independent experiments were performed. The identity of each mouse was blinded to the personal who operated the measurement.

### Immunohistological and immunofluorescent staining

For immunohistological staining, muscle sections were fixed by 3% H_2_O_2_, and blocked for 20 min at room temperature by goat serum (HyClone). Anti-Fbxo32 (ECM Bioscience, 1:500) antibody was used as primary antibody. Secondary antibodies (Jackson Laboratory) were diluted 1:2000 in blocking buffer and incubated for 1 h at room temperature. Mixture of substrate A (Dako) and B (Dako) were diluted in blocking buffer and incubated for 30 min at room temperature, and developed by DAB+ Chromogen (Dako). Images were acquired by confocal microscopy (Leica). For immunofluorescent staining, cells or sections were fixed with 4% parafamaldehyde and permeabilized with cold methanol, anti-MYH3 (Developmental Studies Hybridoma Bank, clone F1.652, 1:1000), anti-MYHC (Merk Millipore, clone 05-716, 1:1000) and anti-lammnin (Abcam, clone B00648, 1:1000) antibodies were used as primary antibody, respectively. The cells and sections were next stained with Alexa 488-, 561- or 647-labeled anti-mouse or -rabbit antibodies (Invitrogen). All images were acquired by confocal microscopy (Leica).

### Antibodies

Immunoblotting (Supplementary Fig. 15), immunohistological and immunofluorescent stainings for muscle sections and myotubes were performed with the following antibodies: Fbxo32 (ECM Bioscience, AP2041), MYHC (Upstate, 05-715), Laminin (Abcam, ab11575). Immunoprecipitation and immunoblotting assays were performed with the following antibodies: β-catenin (Cell Signaling, 9562 s), Tcf3 (Abcam, ab11176), TBP (Abcam, ab818), RNA Polymerase II (Novous, Nb100-1806), GAPDH (Protein Tech, 60004), Dkk3 (R&D system, AF948).

### ChIP assays

ChIP assays were performed as previously described^63^. Briefly, cells were fixed with 1% formaldehyde for 10 min at room temperature. Nuclei were isolated and chromatins were extracted from the nuclei. The chromatins were then sheared to 200–500 bp by sonication (Qsonica). Antibodies against FoxO3, β-catenin (Cell Signaling), Tcf3 (Abcam), and RNA Polymerase II (Novus) were applied. The DNA immunoprecipitated by the antibodies were detected byr qPCR. The primers used are listed below:

*Fbxo32* for Pol II-F: 5′-ATCCCATACAGAACCCAGGA-3′

*Fbxo32* for Pol II-R: 5′-GAAAGGCAAACAGCCAAGTC-3′

*Fbxo32* for FRE-F, 5′-CGGCCAAAGAACAAGGACTA-3′

*Fbxo32* for FRE-R: 5′-GGTCAGGACCACTCTCTGG-3′

*Trim63 for Pol II-F: 5*′*-TGCTCATCCCTGCATGTGAT*-*3′*

*Trim63 for Pol II-R: 5*′-*GGCCTCAGACCAAGACAGAAGT-3′*

*Trim63 for FoxO-F: 5*′-*TATCTGGCTCTCCCCTGAAC-3′* (adopted from)^11^

*Trim63 for FoxO-R: 5*′*-CCTCAAAGATTTGGCCCTCT-3′* (adopted from)

*Axin2*-F, 5′-CCCGGGTTCTTTCTCCAGTA-3′

*Axin2*-R, 5′-CCCAGGGCAAAGTAATCCAA-3′

*Ccnd1*-F, 5′-GGTAACTGGCACACACAACC-3′

*Ccnd1*-R, 5′-GGACTGTCAATCACCGCATC-3′

### Myofiber diameter and cross section area measurement

Myofiber diameter was measured by Adobe Acrobat 9 pro software (Adobe). Three independent visual fields in each sample were chosen randomly. Three-hundred fibers from each visual field were measured for each treatment. The myofiber cross section area was measured by Image J software (NIH). Three independent visual fields in each sample were chosen randomly. Three-hundred myofibers from each visual field were measured for analysis. The identity of the samples was blinded to the personnel who performed the measurement.

### Statistical analysis

Two-tails student’s test was performed for pairwise comparison among groups. Statistical significance was set at *p* < 0.05.

### Data availability

The complete mRNA-seq data were uploaded to the GEO (Gene Expression Omnibus, http://www.ncbi.nlm.nih.gov/geo) database with the accession number GSE78147.

## Electronic supplementary material

Supplementary information
