# Supplementary material for: Dkk3 dependent transcriptional regulation controls age related skeletal muscle atrophy
Source: Nat Commun. 2018 May 1;9:1752. doi: 10.1038/s41467-018-04038-6 (PMC5931527; doi:10.1038/s41467-018-04038-6)
Supplement: Supplementary file 1 — Supplementary information [file 41467_2018_4038_MOESM1_ESM.pdf]

a

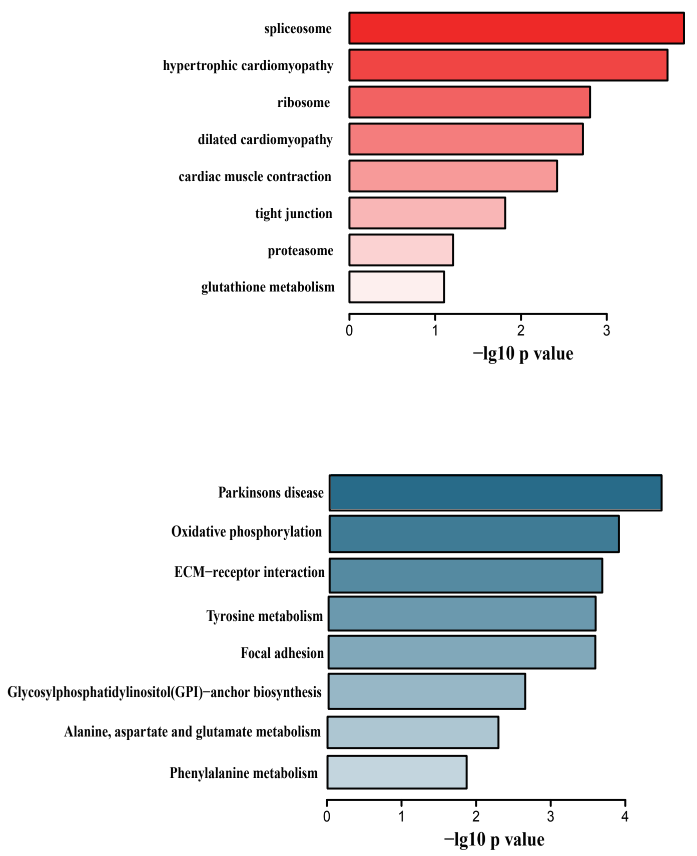

b

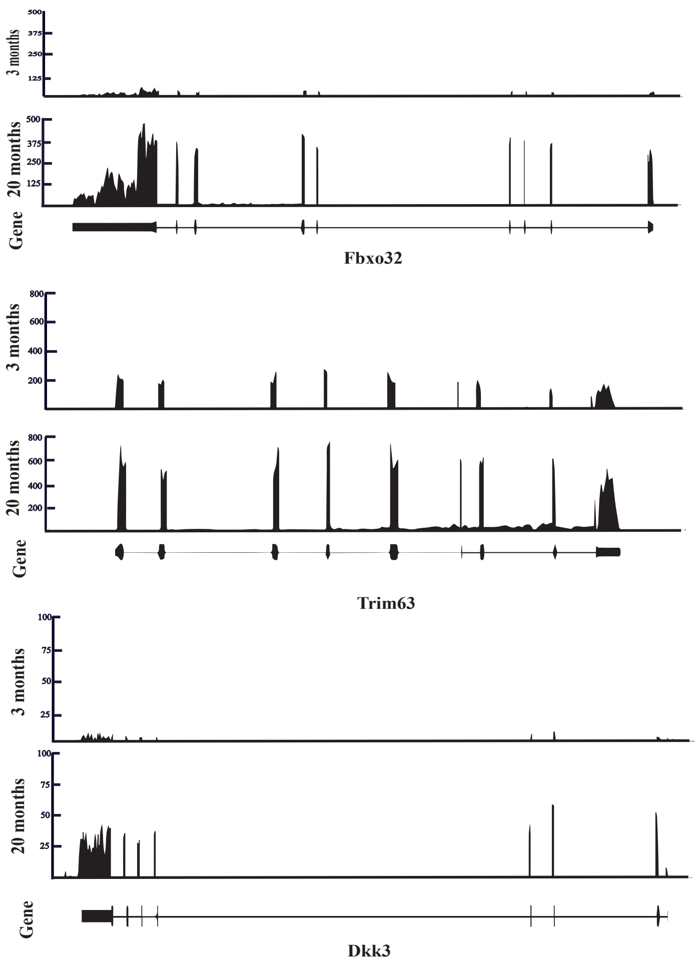

c

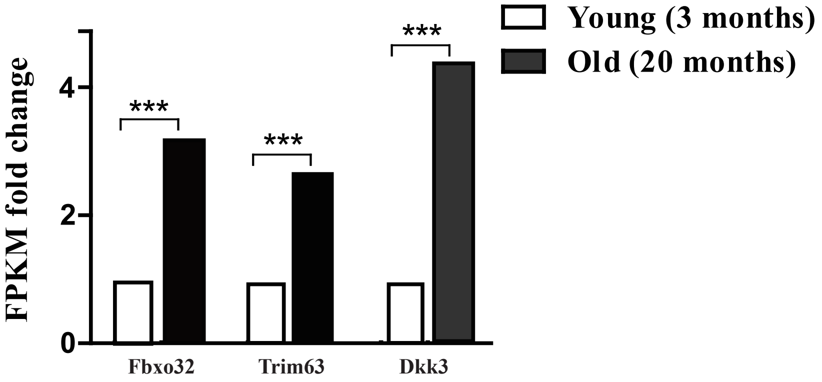

Supplemental Figure 1. Gene ontology analysis of the genes showing significant changes in old (20 months) muscles. (a) Significantly changed genes were picked from the mRNA-seq data followed by KEEG pathway analysis using DAVID software. Top 8 groups were shown. Red indicated the genes up-regulated in old (20 months) muscles, blue indicated down-regulated genes in old (20 months) muscles. p value was calculated by the Benjamini-Hochberg test.(b) Peak distribution of Fbxo32, Trim63 and Dkk3. The peak intensity is significantly higher in old muscles. (c) FPKM fold changes for Fbxo32, Trim63 and Dkk3 in old (20 months) and young (3 months) muscles. ** indicated p< 0.01, *** indicated p< 0.001. p value calculation was based on two-tail t test.

a

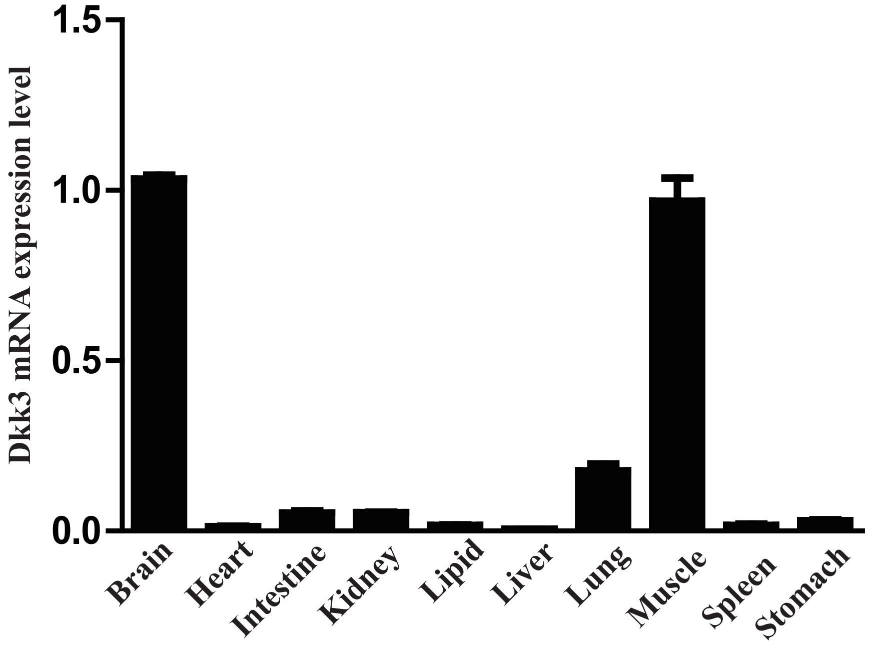

b

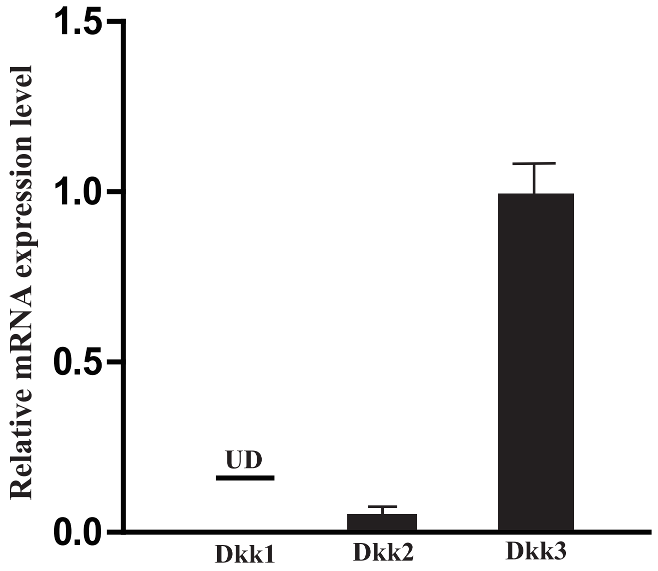

Supplemental Figure 2. Dkk3 is enriched in muscle and brain. (a) RT -qPCR analysis was performed to evaluate the abundance of Dkk3 in various tissues. Error bars indicated standard deviation and were based on 3 independent experiments. (b) RT-qPCR analysis was performed to survey the expression levels of Dickkopf family members Dkk1, 2, and 3. Error bars indicated standard deviation and were based on 3 independent experiments. ND indicated undetectable.

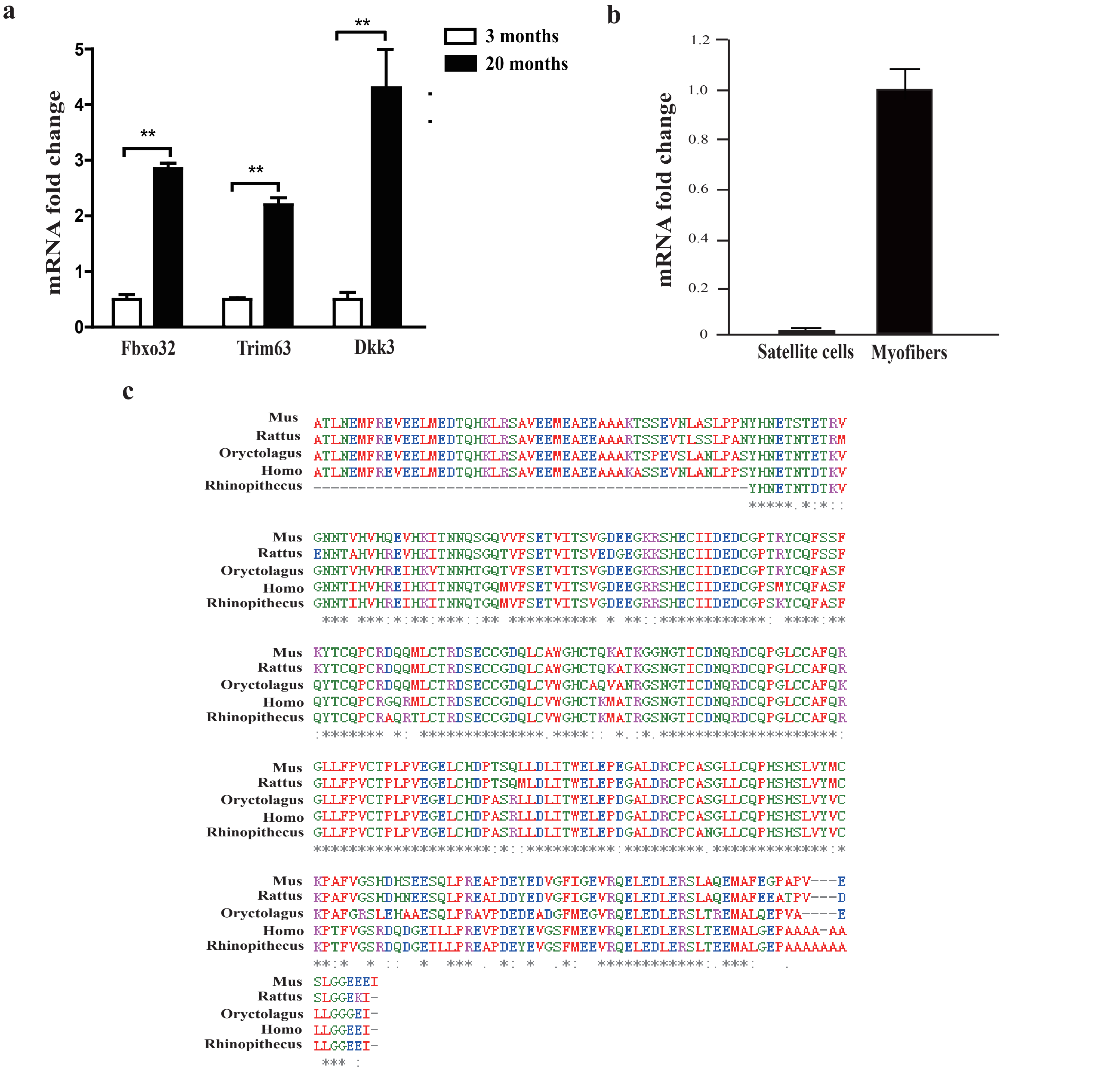

Supplemental Figure 3. Dkk3 was up-regulated in old muscles. (a) The expression levels of Fbxo32, Trim63 and Dkk3 were assayed by RT-qPCR in TA muscles derived from young (3 months old) or old (20 months old) mice to confirm the mRNA-seq analysis results. Error bars indicated standard deviation and were based on 3 independent experiments. ** indicated p<0.01 based on two tail t test. (b)The expression level of Dkk3 is low in aged satellite cells. Satellite cells were isolated from 20 months old mice by FACS sorting as previously described (Fu et al, 2015, Cell Research, 25(6):655-73.). The expression levels of Dkk3 were examined by RT-qPCR. (c) Amino acid sequence similarity comparison between Dkk3 proteins from different species using CLUSTALW2 software. * indicated identical amino acids. : indicated similar amino acids.

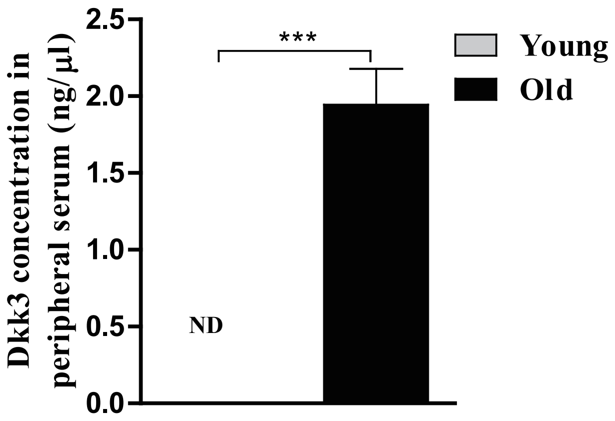

Supplemental Figure 4. Dkk3 concentration was up-regulated in peripheral blood in old mice. ELISA assays were performed to measure the circulating Dkk3 concentration with serum from young adult (3 months) or old (20 months) mice. Error bars indicated standard deviation and were based on 10 independent experiments. ND indicated not detected; *** indicated $p<0.001$.

Yin et al Supplemental Figure 4

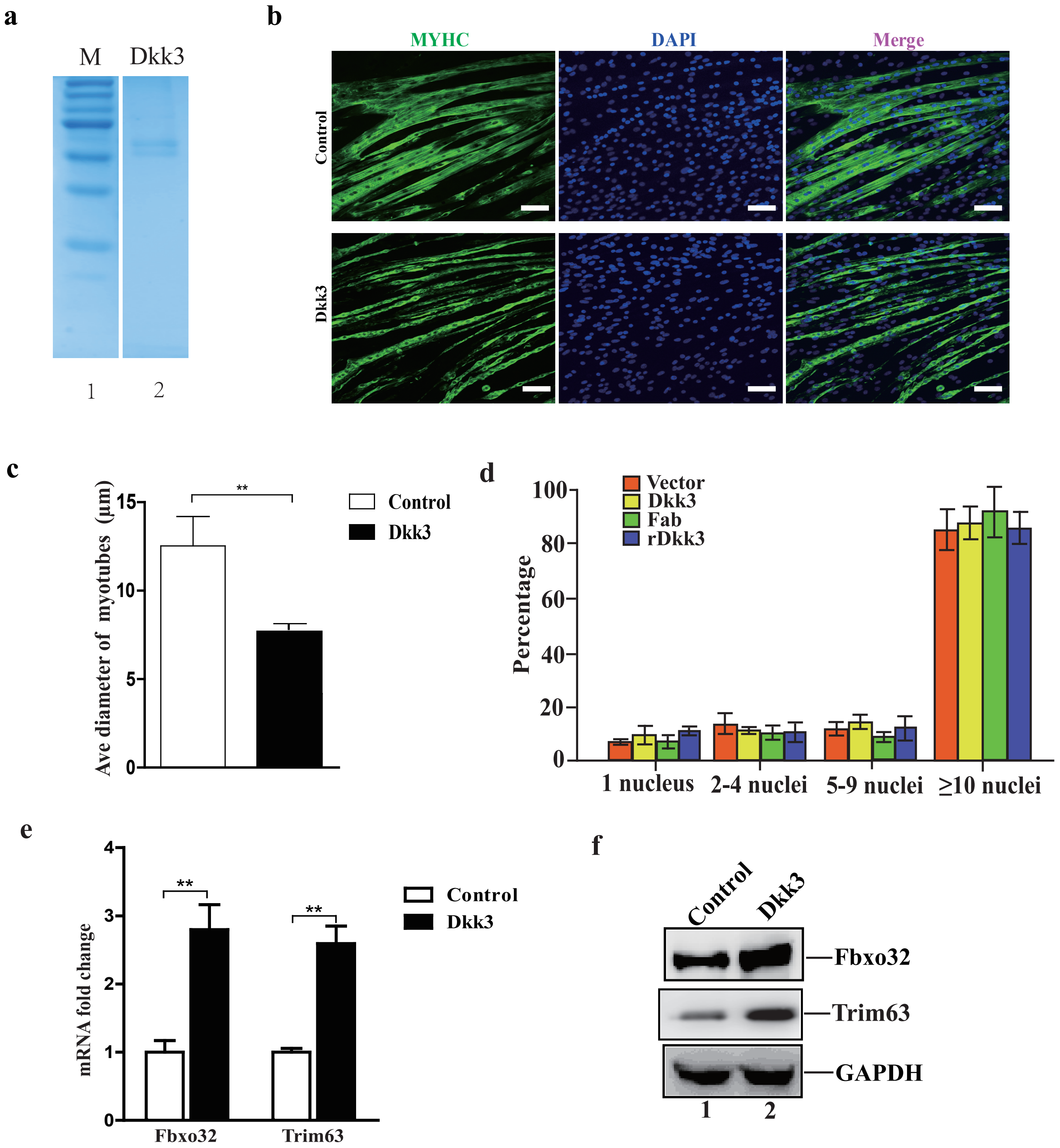

Supplemental Figure 5. Supplementation of recombinant Dkk3 to primary myotube cultures induced atrophy. (a) Commassie blue staining of the purified recombinant Dkk3 protein. Lane 1 indicated molecular weight marker. Lane 2 indicated purified Dkk3. (b) Representative images of immunofluorescent staining of primary myotubes treated with recombinant Dkk3. Irrelative recombinant protein was added as control. Green indicated MYHC staining; DAPI indicated nuclei staining; merge indicated the merged images of MYHC and nuclei staining. Scale bars, 50µm. (c) The average diameters of primary myotubes treated with Dkk3 or irrelative control protein. Error bars indicated standard deviation and were based on 3 independent experiments. ** indicated $p<0.01$ based on two tail t test. (d) Fusion index of myotubes infected by vector or Dkk3 or myotubes treated with Fab (control) or recombinant Dkk3 (rDkk3). (e) The mRNA level of Fbxo32 and Trim63 in primary myotubes treated with Dkk3 or irrelative proteins. Error bars indicated standard deviation and were based on 3 independent experiments. ** indicated $p<0.01$ based on two tail t test. (f) The protein levels of Fbxo32 and Trim63 in primary myotubes treated with Dkk3 or irrelative proteins as illustrated by immunoblotting. Lane 1 indicated proteins extracted from myotubes treated with irrelative protein. Lane 2 indicated proteins extracted from myotubes treated with recombinant Dkk3. GAPDH serves as internal control.

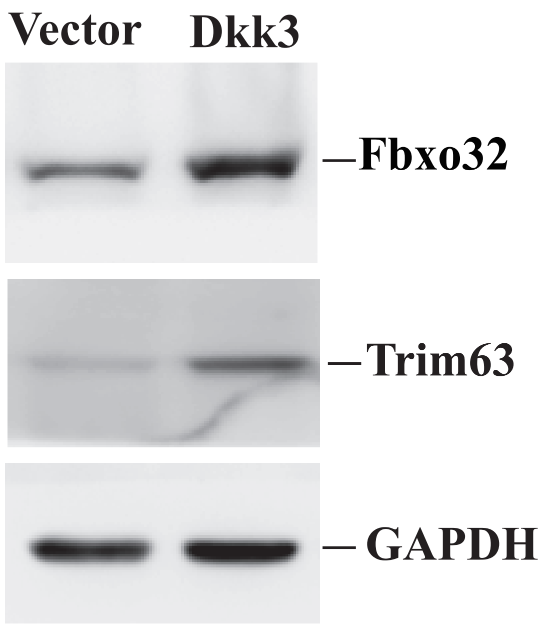

Supplemental Figure 6. Fbxo32 and Trim63 protein levels were up-regulated after ectopic Dkk3 expression. A small portion of TA muscles were taken to make protein extracts. The protein extracts were subjected to anti-Fbxo32, anti-Trim63, and anti-GAPDH immunoblotting.

Yin et al Supplemental Figure 6

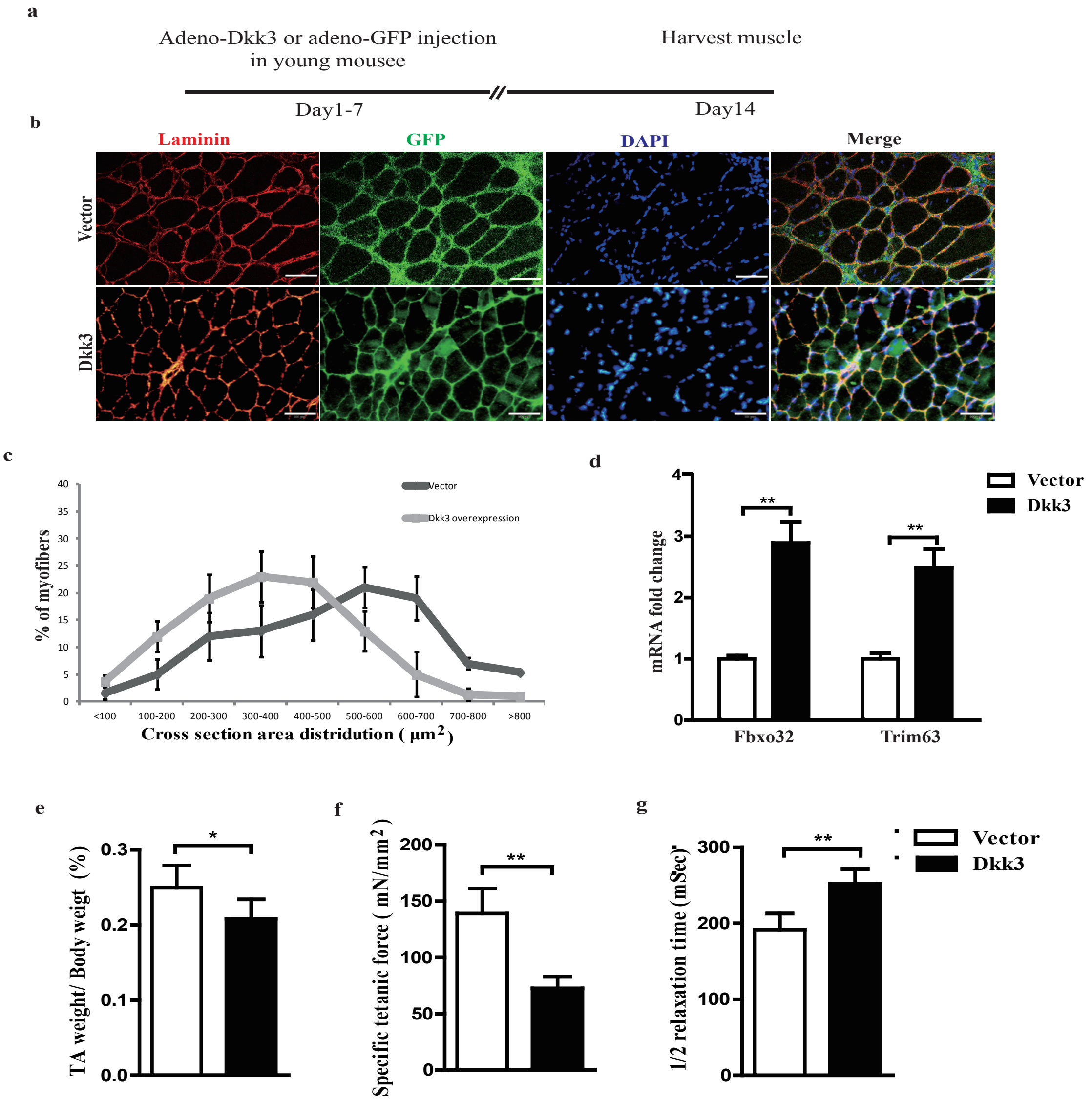

Supplmental Figure 7. Over-expression of Dkk3 in vivo induced muscle atrophy in young mice. (a) Adenovirus encoding Dkk3-GFP was injected to TA muscles in young mice (3 months) intramuscularly. Adenovirus encoding GFP vector was injected to the TA muscle at the opposite side of the same mouse as control. Injections were performed once a day for 7 continuous days. TA muscles were harvested at day 14 (1 week after the last injection) for further analysis. (b) Immunofluorescent staining images of muscle cross sections derived from TA muscles over-expressing Dkk3 or vector control. Red indicated laminin; Green indicated GFP; DAPI indicated nuclei stained with DAPI; Merge indicated the merged image of lamimin, GFP, and DAPI staining. Scale bars, 50 μm. (c) Percentage distribution of muscle fiber cross section area derived from muscles over-expressing Dkk3 or GFP. Black dots indicated muscle sections with ectopic Dkk3 expression. Grey dots indicated muscle sections expressing vector. Error bars indicated standard deviation and were based on 5 independent experiments. (d) Fbxo32 and Trim63 mRNA levels were measured by RT-qPCR in TA muscles with ectopic Dkk3 expression. Error bars indicated standard deviation and were based on 5 independent experiments. ** indicated $p<0.01$. (e) Weight of TA muscles overexpressing Dkk3 was compared to those expressing vector control. Error bars indicated standard deviation and were based on 5 independent experiments. * indicated $p<0.1$. (f) Specific titanic force of TA muscles over-expressing Dkk3 or vector control in young mice (3 months). Error bars indicated standard deviation and were based on 5 independent experiments. ** indicated $p<0.01$. (g) 1/2 relaxation time of TA muscles overexpressing Dkk3 or vector control in young mice (3 months). 1/2 relaxiation time of TA muscles in old mice were compared that young mice (3 months). Error bars indicated standard deviation and were based on 5 independent experiments. ** indicated $p<0.01$.

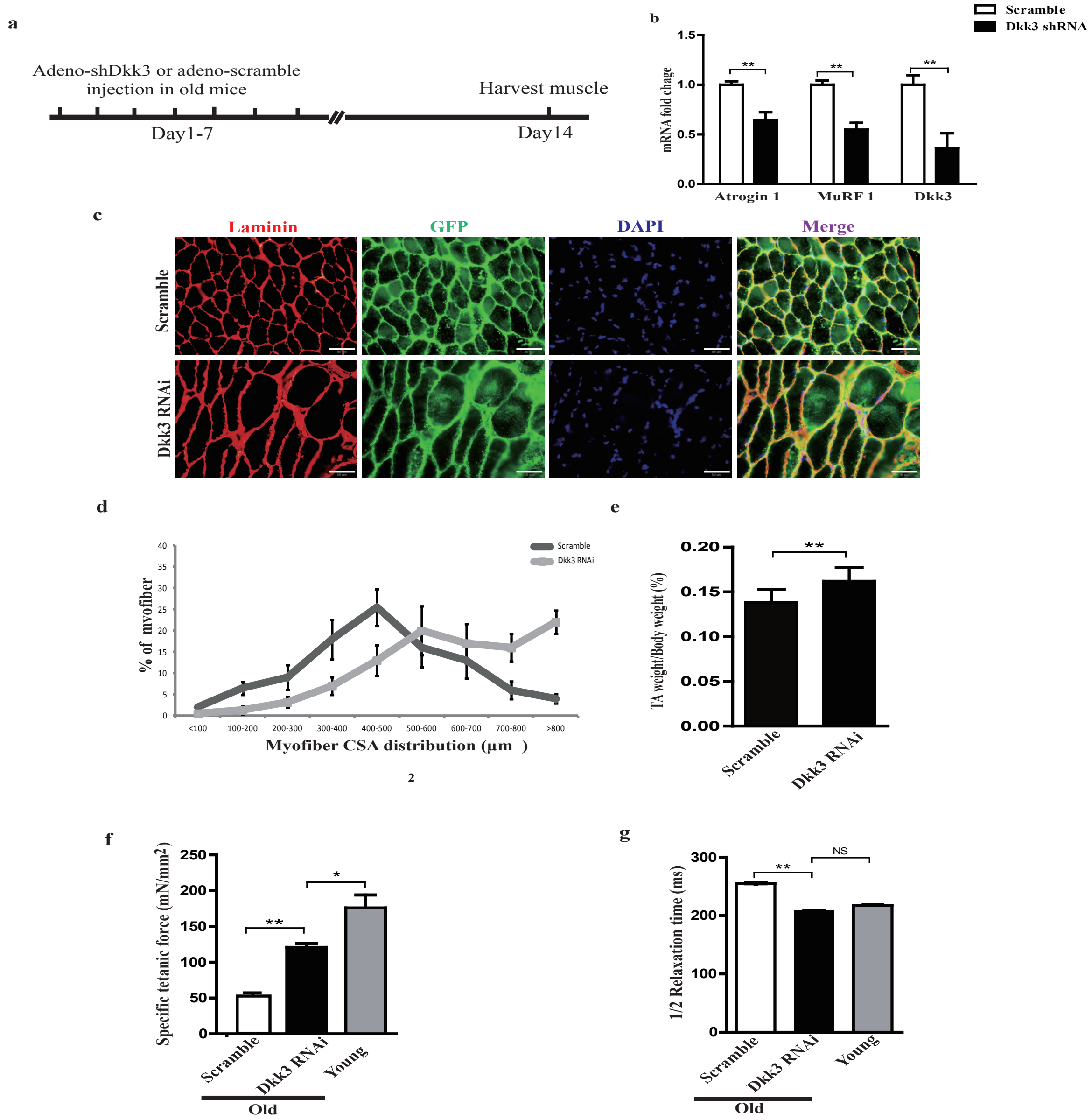

Supplemental Figure 8. Reduction of Dkk3 level rescued age related muscle atrophy. (a) Adenovirus encoding shRNA targeting Dkk3 was injected to TA muscles in aged mice (20 months) intramuscularly. Adenovirus encoding scramble shRNA was injected to the TA muscle in mice with the same age and gender. Injections were performed once a day for 7 continuous days. TA muscles were harvested at day 14 (1 week after the last injection) for further analysis. The shRNA against Dkk3 reduced Dkk3 expression efficiently as indicated by bars labeled with Dkk3. Dkk3 RNAi led to reduction of Fbxo32 and Trim63 as indicated by the bars labeled with Fbxo32 and Trim63. Error bars indicated by standard deviation and were based on 5 independent experiments. ** indicated $p<0.01$. (b) Representative immunofluorescent staining images of muscle cross sections derived from TA muscles in old mice treated with shRNA against Dkk3 or scramble control. Red indicated laminin staining. Scale bars, 50 µm. (c) Percentage distribution of muscle fiber cross section area derived from muscles treated with shRNA against Dkk3 or scramble control. Black dots indicated muscle sections with Dkk3 RNAi. Grey dots indicated muscle sections with scramble control treatment. Error bars indicated standard deviation and were based on 5 independent experiments. (d) The percentage of TA muscle account for the whole body weight derived from muscles treated with shRNA against Dkk3 or scramble control. Error bars indicated standard deviation and were based on 5 independent experiments. ** indicated $p<0.01$. (e) Specific titanic force of TA muscles treated with shRNA against Dkk3 or scramble control in old mice (20 months). The specific titanic force of TA muscles in old mice were compared that young mice (3 months). Error bars indicated standard deviation and were based on 5 independent experiments. ** indicated $p<0.01$. * indicated $p<0.1$. (g) 1/2 relaxation time of TA muscles treated with shRNA against Dkk3 or scramble control in old mice (20 months). 1/2 relaxiation time of TA muscles in old mice were compared that young mice (3 months). Error bars indicated standard deviation and were based on 5 independent experiments. ** indicated $p<0.01$. NS indicated no significant changes.

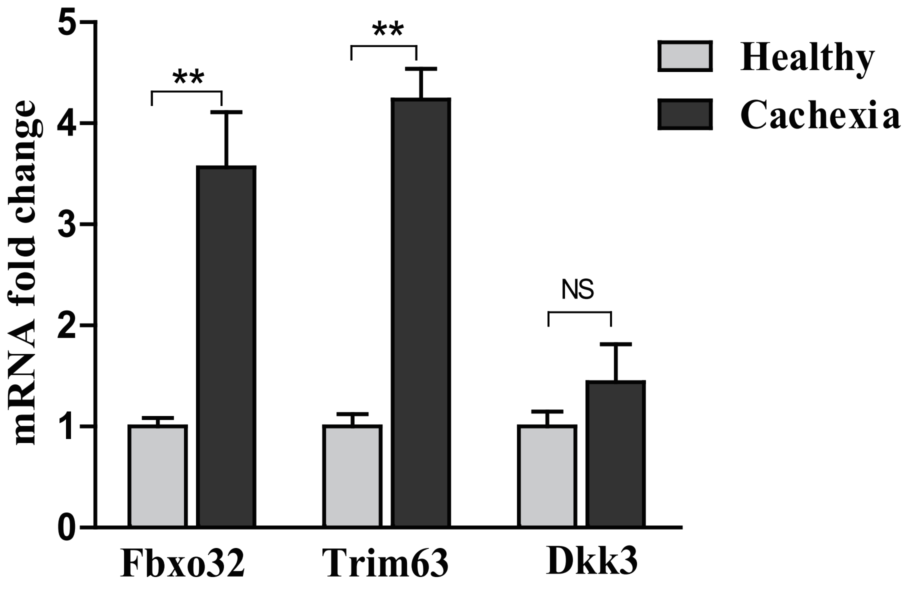

Supplemental Figure 9 Dkk3 expression level did not change in cachetic muscles. TA muscles were harvested from 6 month old mice with or without lung cancer. The mice with lung cancer displayed obvious cachexia. The expression levels of Fbxo32, Trim63, and Dkk3 were examined by RT-qPCR. Error bars indicated standard deviation and were based on 5 independent experiments. ** indicated p<0.01 based on two tail t test.

Yin et al Supplemental Figure 9

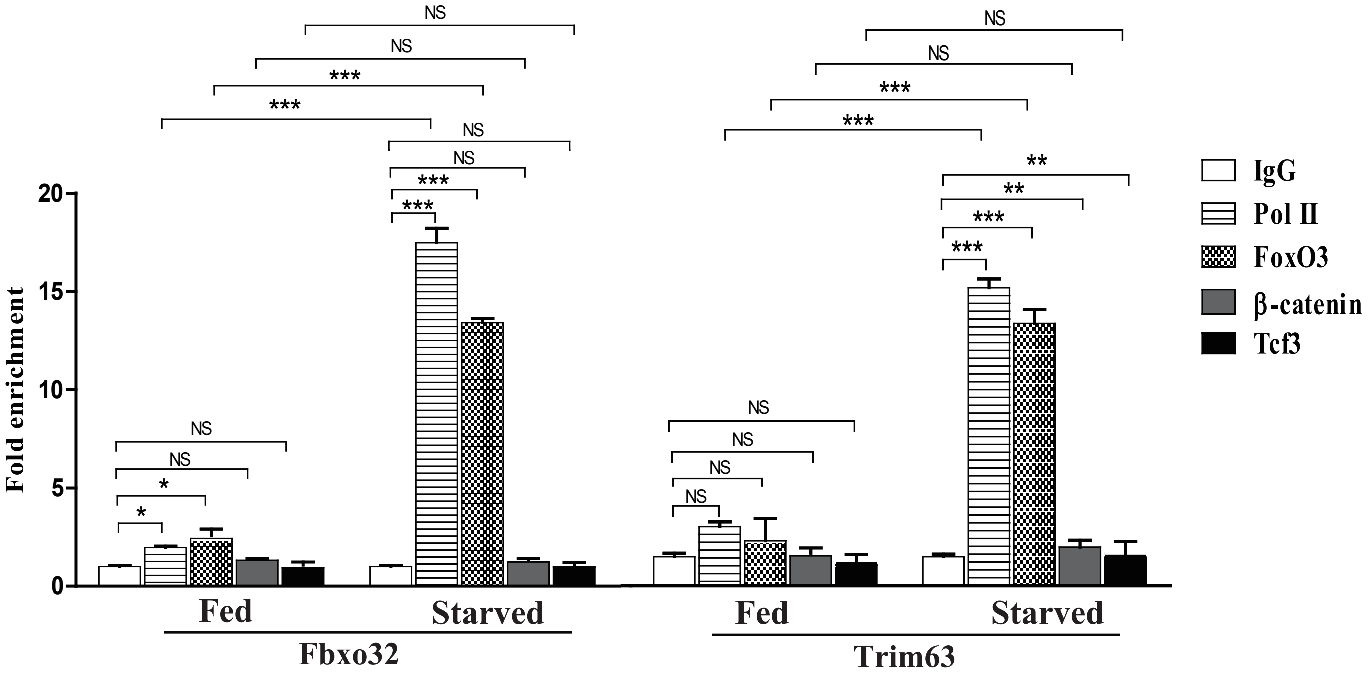

Supplemental Figure 10. FoxO3, but not β-catenin, bound the core promoters of Atrogin 1 and MuRF 1 upon starvation. ChIP analysis of RNA pol II, FoxO3, β-catenin and Tcf3 on the core promoters of Fbxo32 and Trim63 in primary myotubes after starvation. Error bars indicated standard deviation and were based on 3 independent experiments. * indicated $p < 0.05$, ** indicated $p < 0.01$, *** indicated $p < 0.001$. NS indicated not significant. All p values were calculated based on two tail t test.

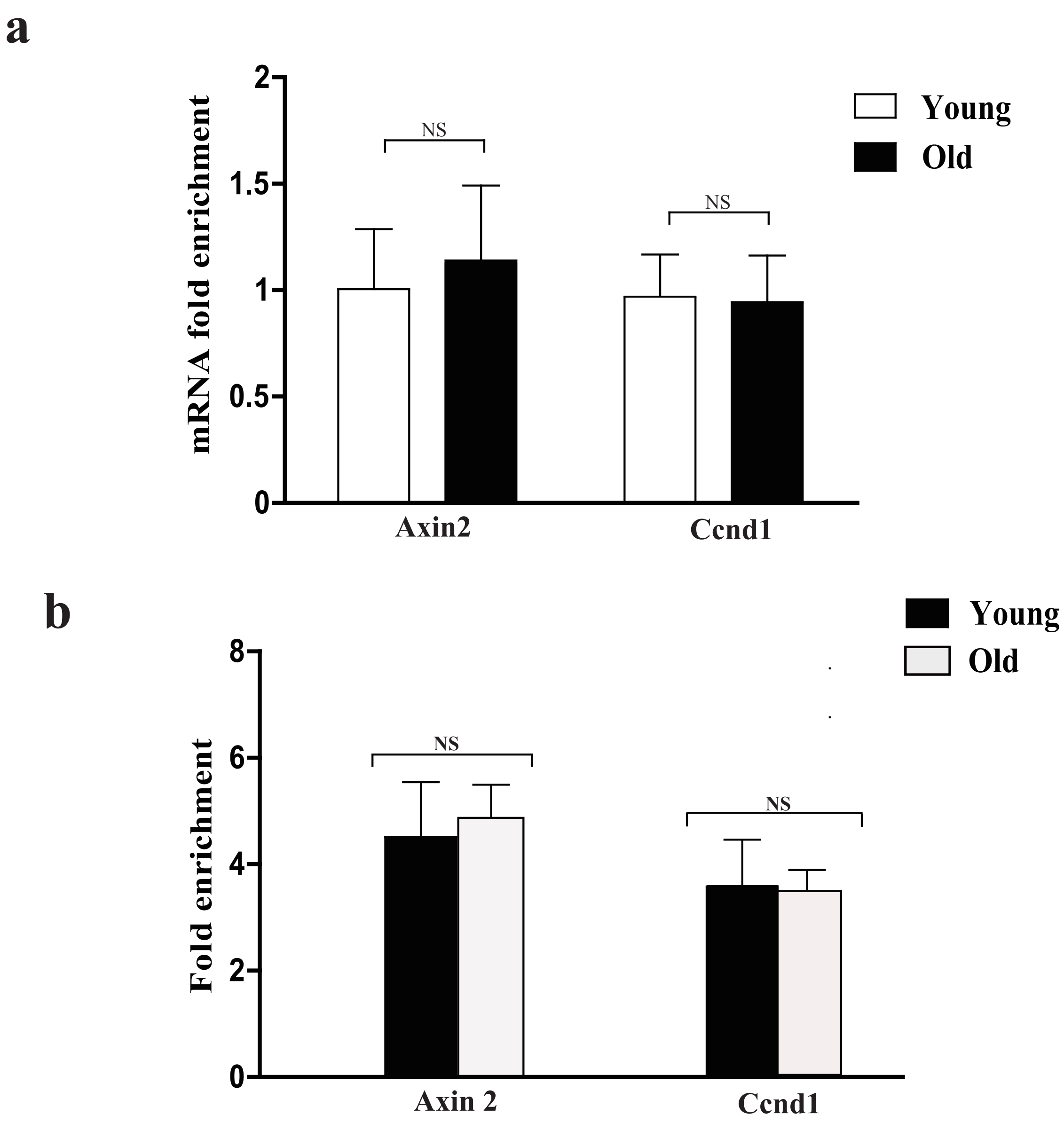

Supplemental Figure 11. The expression of Wnt signaling targets was not affected by enhanced protein-protein interaction between FoxO3 and β-catenin. (a) The mRNA levels of canonical Wnt target gene Axin2 and Ccnd1 were not altered in old muscles (20 months). (b) ChIP analysis of β-catenin in TA muscles from young adult (3 months) or old (20 months) mice. Error bars indicated standard deviation and were based on 3 independent experiments. NS indicated not significant based on two tail t test.

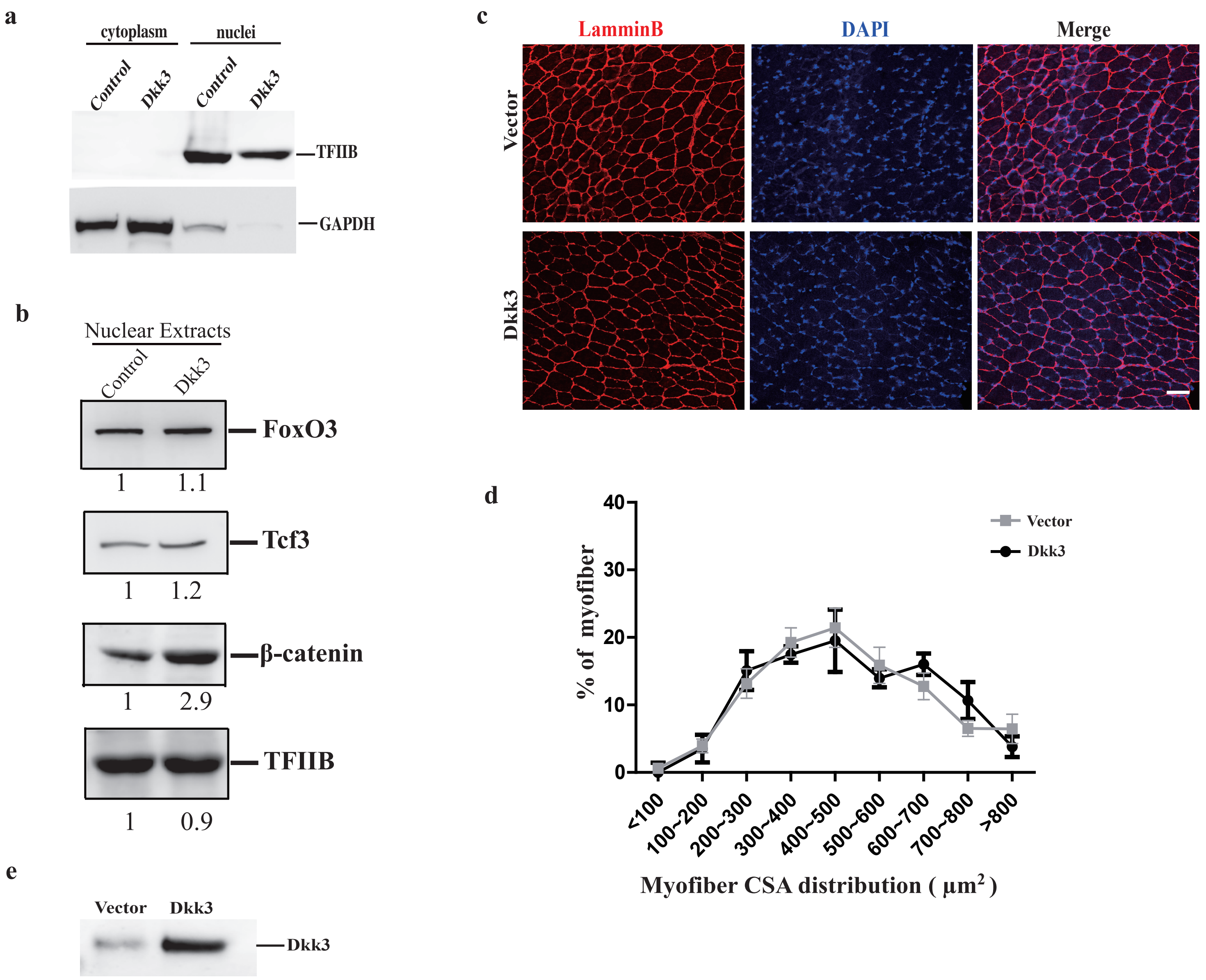

Supplemental Figure 12. FoxO3 was required for Dkk3 induced muscle atrophy. (a) The nuclei of myotubes with or without Dkk3 treatment were isolated, respectively. Anti-TFIIB immunoblotting was performed to indicate the presence of nuclear proteins in the nuclear fraction, but not in the cytoplasmic fraction. Anti-GAPDH immunoblotting was performed to indicate the presence of cytoplasmic protein predominantly in the cytoplasmic fraction.(b) Nuclear extracts prepared from myotubes treated with Dkk3 or the irrelevant control protein were subjected to immunoblotting of FoxO3, Tcf3 and β-catenin. TFIIB immunoblotting served as internal control. The numbers under each blot indicated the quantification of fold increase. (c) Immunofluorescent staining images of muscle cross sections from FoxO3-/- TA muscles infected by Dkk3 or control virus. Red indicated laminin; Blue indicated nuclei staining by DAPI; Merge indicated the merged image of lamimin and DAPI. Scale bars, 50 μm. (d) Percentage distribution of muscle fiber cross section area of FoxO3-/- muscles over-expressing Dkk3 or vector. Black dots indicated muscle sections with ectopic Dkk3 expression. Grey dots indicated muscle sections with vector expression as control. Error bars indicated standard deviation and were based on 5 independent experiments. (e) Whole cell extracts were prepared from FoxO3-/- muscle tissues infected with Dkk3 or vector virus. The extracts were subjected to immunoblotting with Dkk3 antibody.

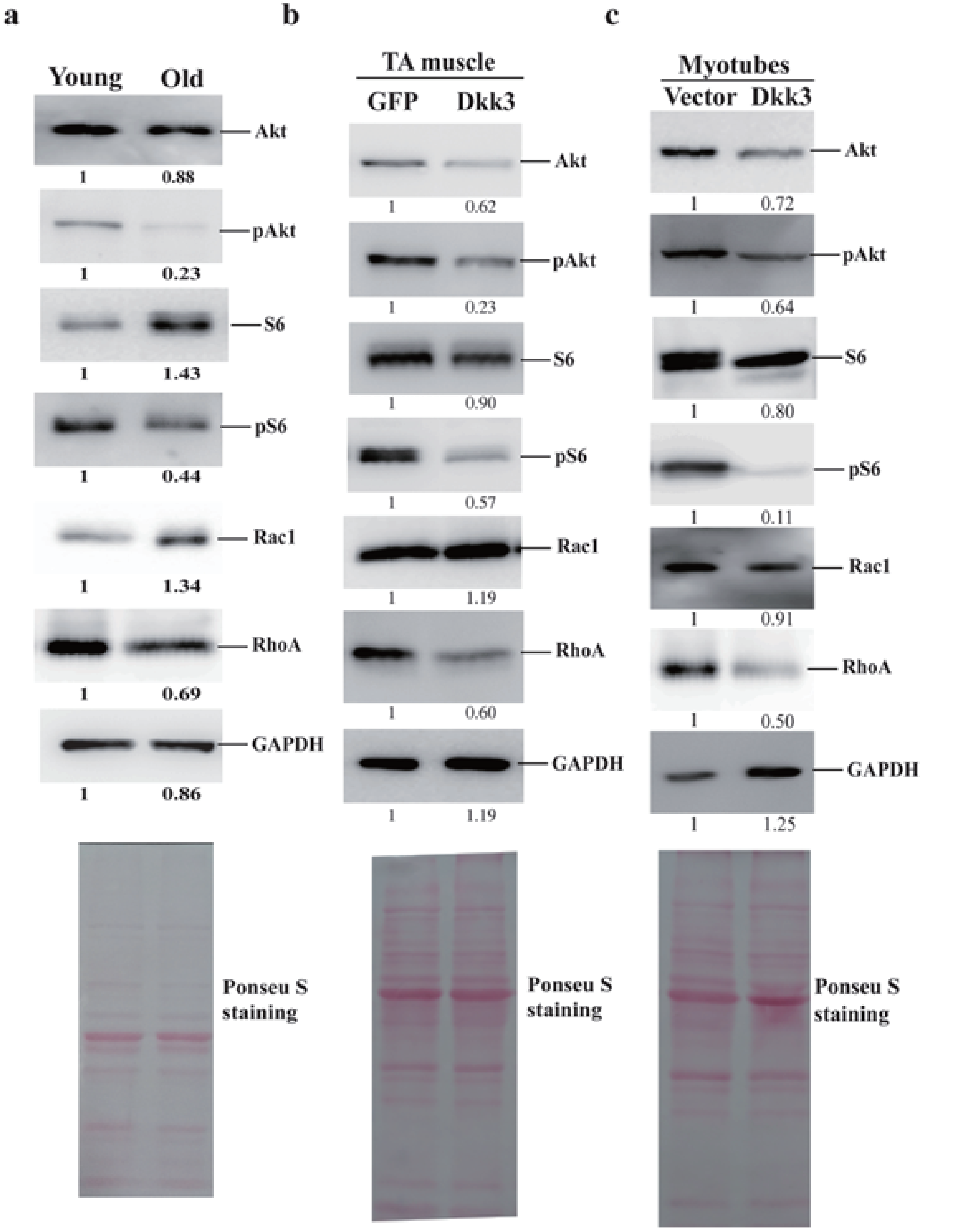

Supplemental Figure 13. Dkk3 treatment inhibits non-canonical Wnt signaling. (a) Immunoblotting of the key components of Akt/mTOR and PCP signaling pathways with whole cell extracts from aged (20 months) TA muscles. (b) Immunoblotting of the key components of Akt/mTOR and PCP signaling pathways with whole cell extracts from TA muscles over-expressing Dkk3. (c) Immunoblotting of the key components of Akt/mTOR and PCP signaling pathways with whole cell extracts from myotubes over-expressing Dkk3. Ponseau S staining indicated equal loading.

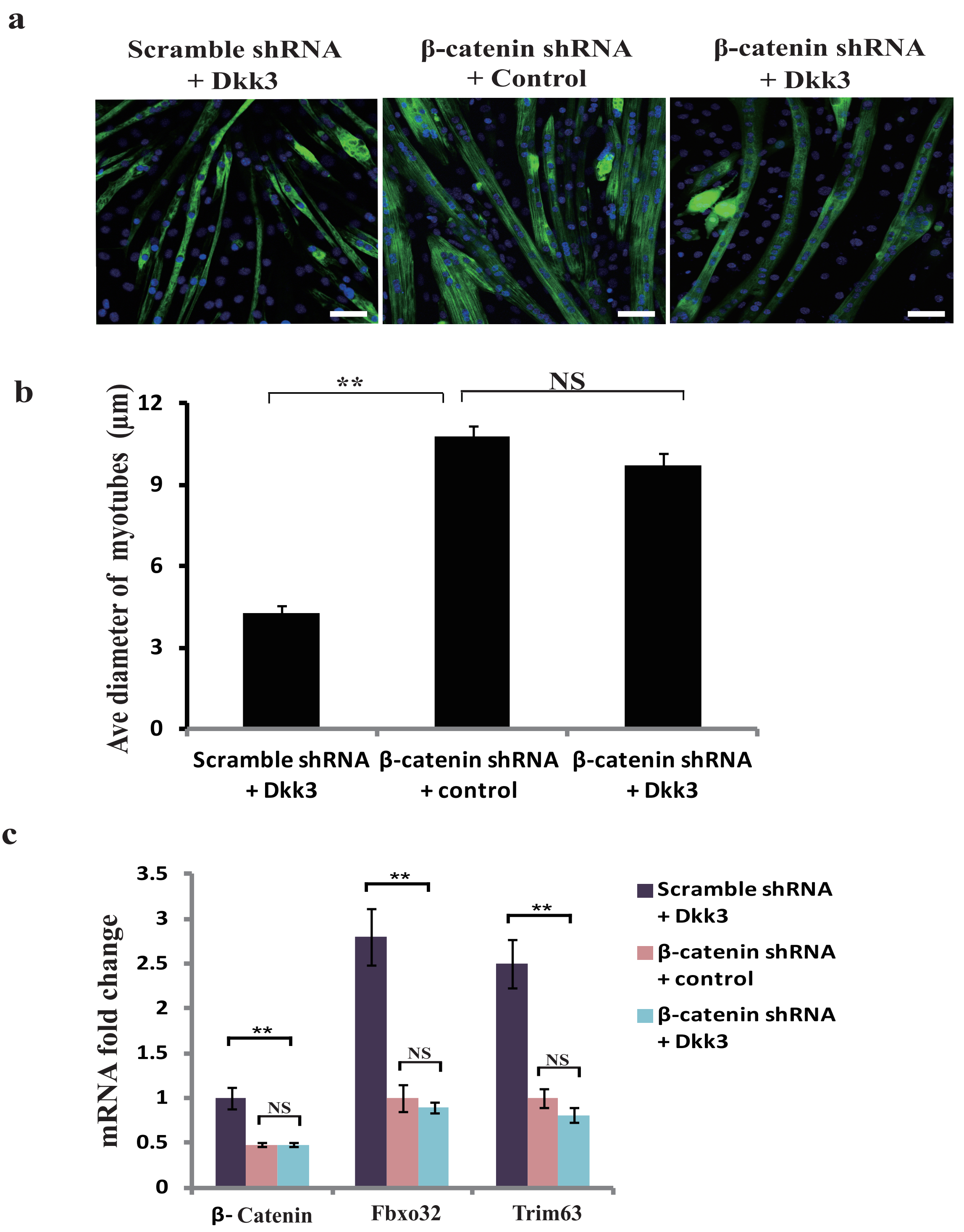

Supplemental Figure 14. β-catenin was required for Dkk3 induced muscle atrophy. (a) Myotubes were infected by adenovirus encoding either scramble shRNA or shRNA targeting β-catenin, respectively. 48 hours after infection, the infected myotubes were treated with either Dkk3 or control protein for 24 hours. Myosin heavy chain immunofluorescent staining were performed to show the shape of myotubes. Green indicated MyHC staining; blue indicated DAPI staining of nuclei. The merged images were shown. Scale bars, 50 μm. (b) The average diameters of myotubes undergone different treatment. Error bars indicated standard deviation and were based on 3 independent experiments. ** indicated $p<0.01$. NS indicated no significant difference. (c) β-catenin expression level decreased after RNAi targeting β-catenin. Fbxo32 and Trim63 expression levels were unchanged upon Dkk3 treatment after β-catenin was knocked down. "Scramble shRNA + Dkk3" indicated myotubes infected with adenovirus encoding scramble shRNA and treated with Dkk3. "β-catenin shRNA + control" indicated myotubes infected with adenovirus encoding shRNA targeting β-catenin and treated with control protein. "β-catenin shRNA + Dkk3" indicated myotubes infected with adenovirus encoding shRNA targeting β-catenin and treated with Dkk3. Error bars indicated standard deviation and were based on 3 independent experiments. ** indicated $p<0.01$. NS indicated no significant change.

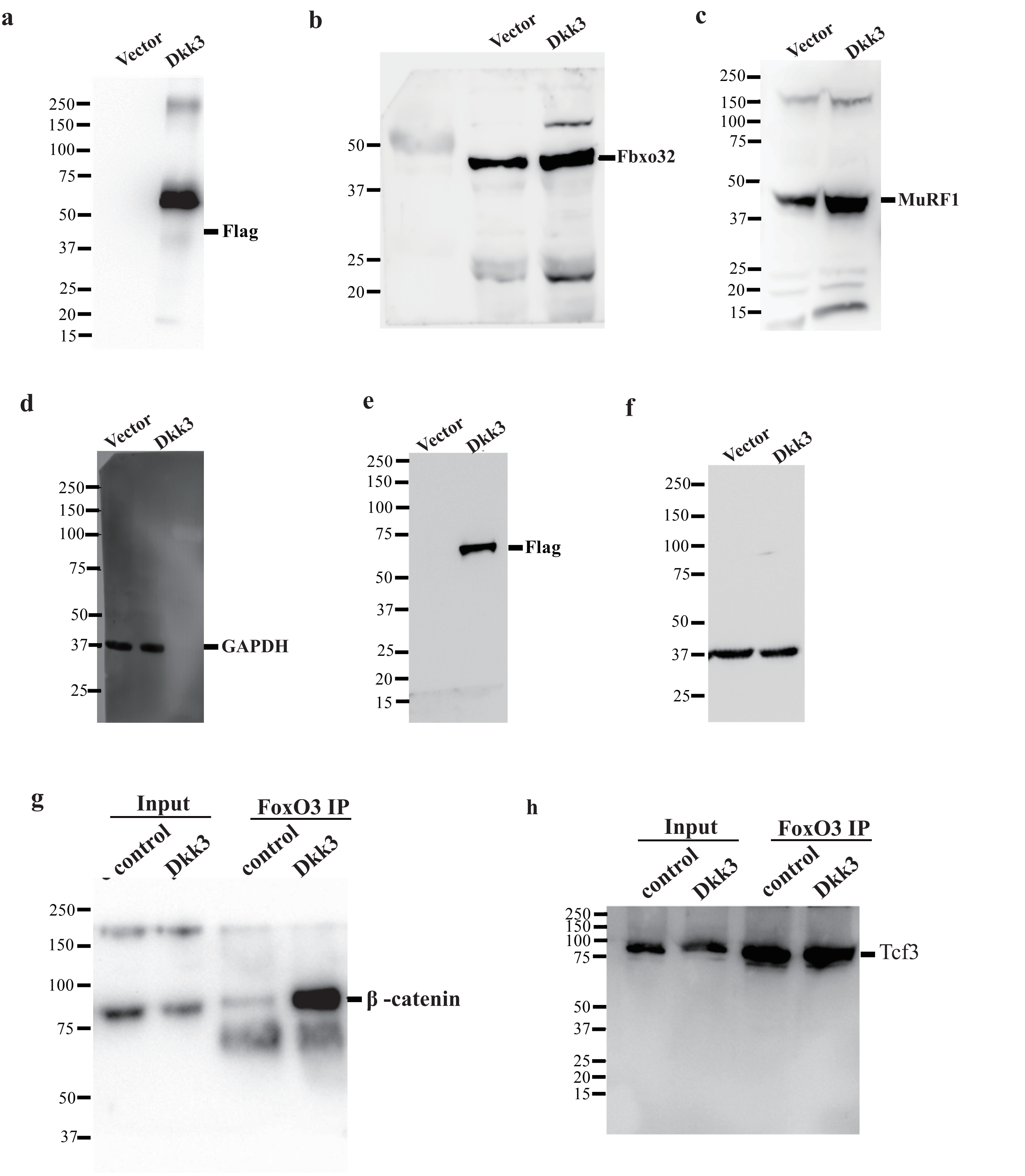

Supplemental Figure 15. a. Full blot of Flag immunoblotting for Figure 2d Flag panel. b. Full blot of Fbxo32 immunoblotting for Figure 2d Fbxo32 panel. c. Full blot of MuRF 1 immunoblotting for Figure 2d MuRF 1 panel. d. Full blot of GAPDH immunoblotting for Figure 2d GAPDH panel. e. Full blot of Flag immunoblotting for Figure 3b Flag panel. f. Full blot of GAPDH immunoblotting for Figure 3b GAPDH panel. g. Full blot of β-catenin immunoblotting in Figure 6d β-catenin panel. h. Full blot of Tcf3 immunoblotting in Figure 6d Tcf3 panel.
